# Supplementary material for: Metabolic responses of rice source and sink organs during recovery from combined drought and heat stress in the field
Source: Gigascience. 2019 Aug 21;8(8):giz102. doi: 10.1093/gigascience/giz102 (PMC6703437; doi:10.1093/gigascience/giz102)
Supplement: giz102_GIGA-D-19-00175_Revision_1 [file giz102_giga-d-19-00175_revision_1.pdf]

GigaScience

Metabolic responses of rice source and sink organs during recovery from combined drought and heat stress in the field

--Manuscript Draft--

**Manuscript Number:**	GIGA-D-19-00175R1
**Full Title:**	Metabolic responses of rice source and sink organs during recovery from combined drought and heat stress in the field
**Article Type:**	Research
**Funding Information:**	German Federal Ministry of Economic Cooperation and Development (11.7860.7-001.00) — Dr. Krishna S.V. Jagadish
**Abstract:**	Background<br><br>Drought and heat stress effects on rice have been extensively studied, in particular during the sensitive flowering and grain-filling stages. However, in the field these stresses usually occur together because reduced transpirational cooling under drought conditions results in increased plant tissue temperature. In addition, environmental stresses are usually transient and the ability to efficiently recover from stress may be at least as important for overall stress tolerance as the direct stress response itself. Nevertheless, nothing is known about recovery mechanisms after drought and heat stress in rice under field conditions.<br><br>Results<br><br>We have used gas chromatography-mass spectrometry (GC-MS)-based metabolomics to elucidate the metabolic responses of flag leaves, flowering spikelets and developing seeds from three rice cultivars differing in their drought and heat tolerance to rewatering after stress in the field. Within 60 h after rewatering, many stress-responsive metabolites returned to their control levels, although recovery was not complete. In addition, control plants showed developmental differences that were revealed by metabolite profiles during 60 h of post-stress sampling, in particular in developing seeds. Correlation analysis identified several metabolites as marker candidates for the stability of grain yield or quality under conditions of combined drought and heat stress.<br><br>Conclusions<br><br>The rewatering responses of stressed plants seemed to be a combination of the reversal of stress effects and reinitiation of development after stress relief. The identified potential markers can be useful in efforts to breed stress-tolerant rice germplasm to ensure food availability under changing climate conditions.
**Corresponding Author:**	Dirk K Hincha<br>Max-Planck-Institut fur Molekulare Pflanzenphysiologie<br>Potsdam, GERMANY
**Corresponding Author Secondary Information:**	
**Corresponding Author's Institution:**	Max-Planck-Institut fur Molekulare Pflanzenphysiologie
**Corresponding Author's Secondary Institution:**	
**First Author:**	Lovely Mae F Lawas
**First Author Secondary Information:**	
**Order of Authors:**	Lovely Mae F Lawas
	Alexander Erban
	Joachim Kopka

Powered by Editorial Manager® and ProduXion Manager® from Aries Systems Corporation

	Krishna S.V. Jagadish
	Ellen Zuther
	Dirk K Hincha
**Order of Authors Secondary Information:**	
**Response to Reviewers:**	Reviewer reports:<br>Reviewer #1: 1) Are the methods appropriate to the aims of the study, are they well described, and are necessary controls included?<br><br>Experimental rational, methods, scripts and datasets are well descibed and links provided for the research community. Overall, an exellent manuscript for an important research question and the authors have a novel approach. The complexity of metabalomic data (and selection of adequate controls) makes interperation of this type of data extremely difficult. However, the authors address these concerns in their discussion, tempered expectations, and have not over interperated their results.<br><br>2) Are the conclusions adequately supported by the data shown?<br><br>The conclusions the authors have drawn are supported by their data and the statistical analysis they have performed. Further work as suggested by the authors to examine the effect of these metabolites in response to combined heat and drought stress. Both heat and drought are complex abiotic stresses and I would expect metabalomic results to vary, especially when working in field across years rather than under controlled enviroments.<br><br>3) Please indicate the quality of language in the manuscript. Does it require a heavy editing for language and clarity?<br><br>The manuscript is clear and well written.<br>Only one mistake I could find during proof reading:<br>377 stress. Obviously, {T}his hypothesis needs further testing with a larger panel of genotypes.<br>Response: We thank the reviewer for the careful reading and for pointing this out. We have made the necessary correction.<br><br>4) Are you able to assess all statistics in the manuscript, including the appropriateness of statistical tests used?<br><br>From the description in this manuscript, previous experience with PCA and brief look at the source code provided, the analysis seems solid. More documentation in the source code would excellent for others doing similar work.<br><br>Recommendation:<br><br>This manuscript is well founded and well written. The lack of similar studies (as described by the authors) warrents the acceptance of this paper for the research community.<br>This manuscript is of interest to others working in metabolomics in under field conditions and would stimulate the use of this approach into field trials (If this approach can be scaled up).<br><br><br>Reviewer #2: The manuscript titled "Metabolic responses of rice source and sink organs during recovery from combined drought and heat stress in the field" investigated the plant recovery mechanisms after drought and heat stress. The study aim is clear and well designed. The data analysis is rigorous, and the conclusion drawn are supported from the results.<br>Nevertheless, I would suggest reviewing and modify some details that are given below:<br><br>Abstract<br>-Line 43-44 and line 56: the authors stated that the identified metabolites might be useful to ensure FOOD SECURITY under climate changing conditions. According to the FAO definition, food security encompasses different aspects related to food, such

	as availability, access, utilization, stability and safety. However, I think that the results of this study are useful only in ensuring food availability under climate changing conditions, there is no improvement from the safety point of view. Therefore, I would suggest changing "food security" for "food availability".<br>Response: We agree with the reviewer and have changed the phrase as suggested in both places.<br><br>Data description<br>-Line 108-111: I would suggest moving this paragraph to the result section<br>Response: We would rather leave this information under Data description. We think that it is important at that point in the paper, because it provides the reader with the background necessary to understand the rational of the experiments. Also, these are not new results, we simply cite an earlier paper.<br><br>-Line 112: the authors stated that overall they collected 1241 samples, but I cannot understand how to get this number. How many samples/per years? How many samples/per time points? How many biological replicates?<br>Response: We have included a more detailed description of the sample and replicate numbers in Methods/Sample collection now to clarify this point.<br><br>Analysis and discussion:<br>-Line 134: Did the author observed any influence of the harvesting years on the PCA plot?<br>Response: There were differences in metabolite composition between years as conditions in the field are never the same in different years. However, we did not analyse these differences further, but rather treated the samples from all years as replicates to obtain robust metabolic responses. We have therefore not elaborated on the point of yearly variation in our paper.<br><br>-The authors focused on the primary metabolism changes. However, lipids are well known to be involved in the plant response to stresses. May the authors comment on that?<br>Response: We agree that lipids, along with secondary metabolites and other compound classes may also be of importance. However, since we have no data on these other compound classes it did not seem appropriate for us to speculate on that.<br><br>Methods<br>-Line 461-462: three to five replicates. What influence the number of collected replicates per sample? Did the authors analyse all the collected sample replicates?<br>Response: Yes, we analysed all replicates we collected. In most cases we obtained 5 replicates per year, giving us 15 replicates in total across the three years. In some cases it was 4 and in only a few cases it was 3. This is now stated explicitly under Methods/Sample collection to clarify this point. We do not think that the small fraction of samples with less than 14 replicates in total (only about 7%) had any influence on our analyses or the interpretation of the results.
**Additional Information:**	
**Question**	**Response**
Are you submitting this manuscript to a special series or article collection?	No
**Experimental design and statistics**<br><br>Full details of the experimental design and statistical methods used should be given in the Methods section, as detailed in our Minimum Standards Reporting Checklist. Information essential to interpreting the data presented should be made available in the figure legends.	Yes

Have you included all the information requested in your manuscript?	
**Resources**<br><br>A description of all resources used, including antibodies, cell lines, animals and software tools, with enough information to allow them to be uniquely identified, should be included in the Methods section. Authors are strongly encouraged to cite Research Resource Identifiers (RRIDs) for antibodies, model organisms and tools, where possible.<br><br>Have you included the information requested as detailed in our Minimum Standards Reporting Checklist?	Yes
**Availability of data and materials**<br><br>All datasets and code on which the conclusions of the paper rely must be either included in your submission or deposited in publicly available repositories (where available and ethically appropriate), referencing such data using a unique identifier in the references and in the "Availability of Data and Materials" section of your manuscript.<br><br>Have you have met the above requirement as detailed in our Minimum Standards Reporting Checklist?	Yes

Powered by Editorial Manager® and ProduXion Manager® from Aries Systems Corporation

Manuscript with DOI
Click here to view linked References
Click here to access/download;Manuscript;Lawas_et_al_recovery_GigaScienc

Metabolic responses of rice source and sink organs during recovery from combined drought and heat stress in the field

Lovely Mae F. Lawas[1], Alexander Erban[1], Joachim Kopka[1], S.V. Krishna Jagadish[2,3], Ellen Zuther[1], Dirk K. Hincha[1,*]

[1]Max-Planck-Institute of Molecular Plant Physiology, D-14476 Potsdam, Germany

[2]International Rice Research Institute, DAPO Box 7777, Metro Manila, Philippines

[3]Department of Agronomy, Kansas State University, Manhattan, Kansas 66506, USA

[*]Corresponding author:

Dirk K. Hincha, Max-Planck-Institute of Molecular Plant Physiology, Am Mühlenberg 1, D-14476 Potsdam, Germany

e-mail addresses:

LMFL: Lawas@mpimp-golm.mpg.de

AE: Erban@mpimp-golm.mpg.de

JK: Kopka@mpimp-golm.mpg.de

SVKJ: kjagadish@ksu.edu

EZ: Zuther@mpimp-golm.mpg.de

DKH: Hincha@mpimp-golm.mpg.de

Abstract

Background: Drought and heat stress effects on rice have been extensively studied, in particular during the sensitive flowering and grain-filling stages. However, in the field these stresses usually occur together because reduced transpirational cooling under drought conditions results in increased plant tissue temperature. In addition, environmental stresses are usually transient and the ability to efficiently recover from stress may be at least as important for overall stress tolerance as the direct stress response itself. Nevertheless, nothing is known about recovery mechanisms after drought and heat stress in rice under field conditions.

Results: We have used gas chromatography-mass spectrometry (GC-MS)-based metabolomics to elucidate the metabolic responses of flag leaves, flowering spikelets and developing seeds from three rice cultivars differing in their drought and heat tolerance to rewatering after stress in the field. Within 60 h after rewatering, many stress-responsive metabolites returned to their control levels, although recovery was not complete. In addition, control plants showed developmental differences that were revealed by metabolite profiles during 60 h of post-stress sampling, in particular in developing seeds. Correlation analysis identified several metabolites as marker candidates for the stability of grain yield or quality under conditions of combined drought and heat stress.

Conclusions: The rewatering responses of stressed plants seemed to be a combination of the reversal of stress effects and reinitiation of development after stress relief. The identified potential markers can be useful in efforts to breed stress-tolerant rice germplasm to ensure food availability under changing climate conditions.

Keywords: combined stress, drought stress, flowering, grain filling, heat stress, marker metabolites, metabolomics, recovery, rice (*Oryza sativa*)

Background

Plant growth and productivity are threatened by exposure to extreme environmental conditions [1–3]. Temperature and precipitation extremes have resulted, among other climate-related consequences, in heat waves and drought events [4,5] that are projected to continue with increased frequency and intensity in the future [4,6,7]. In parallel, models indicate that high temperature and water scarcity have caused yield losses [8,9], which will be exacerbated under future climate scenarios [10,11]. Rice is among the major crops that have been negatively impacted by drought and heat [12,13], and this poses a serious threat to food availability because rice is a staple food for almost half of the world's population [14].

The effects of heat [15–18] and drought [19–21] on rice have been extensively studied, particularly during the stress-sensitive flowering and grain-filling stages, where they result in significant grain yield and quality losses. Furthermore, the responses of rice to the simultaneous occurrence of these two stresses have been documented [22–26]. Over recent years, an increasing number of studies have focused on the effects of combined drought and heat stress on plants [27,28] due to the recognition that stress combinations are frequent under field conditions and are more detrimental for plants than the single stresses [29]. Yet the molecular mechanisms enabling tolerance to combined drought and heat stress still remain to be elucidated, particularly in cereals [28]. In addition, there is still very little knowledge about the effects of combined stress on plants grown under field conditions.

In most cases, abiotic stresses are transient, with fluctuating temperatures and drought periods followed by rain, and hence plants are subjected to episodes of stress and recovery [30]. Plant survival is in fact determined by both the responses during exposure to stress and during the subsequent recovery phase [31,32]. The extent of recovery depends on the duration and intensity of the stress, and the plant genotype, growth stage and organ/tissue that is examined [33,34]. While the effects of abiotic stresses on plants and the mechanisms by which plants cope with such environmental conditions have been studied in detail, little is known about how plants respond during recovery. In rice, morpho-physiological traits, ABA levels, gene expression and protein levels change during recovery from heat [34,35] and drought [36–38]. In contrast, nothing is known about the recovery process from combined drought and heat stress in rice and there is very limited information about this process in other plant species as well. Most of the stress-induced physiological, biochemical, and metabolic changes observed under stress are reversed upon recovery in eucalypts [39], while combined drought and heat stress induces irreversible changes in water status and chloroplast ultrastructure of tomato leaves [40].

We have conducted experiments to evaluate the responses of field-grown rice to combined drought and heat stress, by withholding water and thus limiting transpirational cooling, and subsequent recovery after rewatering and have reported the effects on agronomic and physiological parameters of three cultivars with contrasting stress tolerance [25]. In addition, we have reported the effects of mild and severe stress treatments on the metabolome of flag leaves, flowering spikelets and developing seeds from the same plants [26]. In the current study, we analyzed the metabolic changes during rewatering following severe drought and heat stress. The objectives of this study were to (i) analyze the metabolite profiles of flag leaves, flowering spikelets, and developing seeds of the three differentially drought and heat tolerant rice cultivars

N22, Dular and Anjali under control, combined drought and heat stress, and rewatering conditions; (ii) compare the metabolite contents of flag leaves and developing seeds collected under fully flooded control conditions on four consecutive days during the early grain-filling stage; (iii) evaluate changes in the content of stress-responsive metabolites in each organ during rewatering at the flowering and early grain-filling stages; and (iv) identify metabolites whose changes in levels between stress and recovery were significantly correlated with reduced grain yield and quality due to combined drought and heat stress.

Data description

Field experiments were performed in three consecutive years (2013, 2014, 2015) during the dry season (flowering and early grain-filling in late April to early May, coinciding with the hottest time of the year) at the International Rice Research Institute (IRRI) in the Philippines. Experiments included the rice cultivars N22 (drought, heat, and combined drought and heat tolerant), Dular (drought tolerant, heat and combined drought and heat susceptible), and Anjali (drought, heat, and combined drought and heat susceptible) [23]. Samples were collected from plants that were either grown under fully flooded control conditions, or were drought stressed during the flowering or early grain-filling stage. At the end of the stress period, plants were rewatered and additional samples were taken 12 h, 36 h and 60 h after rewatering. Drought induced an increase in panicle temperature due to the lack of transpirational cooling, resulting in heat stress [25]. This combined drought and heat stress resulted in significant reductions in grain yield and quality [25]. Samples were taken from flag leaves, flowering spikelets and developing seeds and soluble metabolites were profiled by GC-MS. The data from these 1241 samples have been deposited in the MetaboLights database [41] and are freely available at GigaDB [42].

Details of the metabolite identification and filtering to obtain the final set of metabolites used for detailed analysis are reported in our previous publication [26]. An in-depth analysis of the data from 444 samples obtained under well-watered control conditions, during the early, mild stress phase and during the late, more severe stress phase has been presented recently [26]. Here, we analyzed the metabolomic responses of the plants to rewatering after exposure to severe drought and heat stress. This analysis comprises the same sets of metabolites that were obtained by GC-MS analysis (81 in flag leaves, 88 in flowering spikelets and 67 in developing seeds) in our previous study. We analyzed data from 1151 samples that were obtained under control conditions, during severe drought and heat stress and 12 h, 36 h, and 60 h after rewatering. The 90 samples that were collected during the early, mild stress phase, which preceeded the severe stress, were not considered. We identified metabolites that were significantly changed in their abundance after rewatering compared to the severe stress situation and correlated these changes with either the reduction in yield or the loss of grain quality under stress. These metabolites constitute potential metabolic markers that may be used for the breeding of new stress-tolerant rice cultivars.

Analysis and discussion

Tissue samples of flag leaves during the flowering and early grain-filling stages, flowering spikelets, and developing seeds of the rice cultivars N22, Dular, and Anjali were separately subjected to principal component analysis (PCA) (Fig. 1). In all cases, we observed separation between cultivars and among the treatments, which are described in detail below.

Metabolic profiles change over three days under control conditions during the early grain-filling stage

During early grain filling, samples from plants under control conditions were collected in parallel to the samples collected from the plots designated for stress treatment, starting from the final stress time point until 60 h after rewatering. Due to the set-up of the experiments, it was not possible to obtain similar control samples also during the flowering stage, where we only collected control samples once during the peak of flowering. The control samples collected during the early grain-filling stage may thus represent a developmental time series, although we need to stress that we did not obtain any data independent of the metabolite profiles that allow the characterization of developmental differences. However, the prediction of metabolic differences associated with time-dependent development is substantiated by the PCAs that show shifts along principal component (PC) 1 for the different control samples from flag leaves and developing seeds (Fig. 1B and D, respectively). In fact, 45 metabolites from flag leaves (Fig. 2) and 57 metabolites from developing seeds (Fig. 3) showed significant differences between control samples collected at the final stress time point and at least one of the rewatering time points in any of the three cultivars. This constitutes 56% and 85% of the metabolites analyzed in these organs. It should, however, be noted that many of these metabolites only showed significant differences in content over time in one cultivar and often only at one or two time points (Fig. 2 and 3). Nevertheless, there was a clear tendency in both organs that the content of most metabolites decreased over time. These strong differences in metabolite profiles over a relatively short time span of 60 h under control conditions emphasize the difficulty of defining the best control time points to compare stress treatments to, as the final conclusions will obviously be influenced by this choice. In particular when stressed plants exhibit slower

development compared with the control plants, even samples taken at the same time point may not be an ideal choice and there may in fact not be a single “correct” control.

In flag leaves 11 metabolites (Asn and the bottom 10 metabolites in Fig. 2) showed a general decrease in content in all cultivars across the time points, although these reductions were not always statistically significant. Several other metabolites only showed a reduction in Dular and/or Anjali at the last sampling time point (60 h; 11% of all 57 significantly changed metabolites in N22, 36% in Dular, 42% in Anjali). The majority of metabolites in developing seeds that showed significant changes over time, as grains developed and filled with starch, exhibited reduced levels in all three cultivars (Fig. 3). Interestingly, in developing seeds many significant changes in metabolite content, in particular in Dular, were already evident in samples collected 12 h after the first control samples, when the flag leaf metabolome showed only a few significant changes (Fig. 2). In addition, at the 60 h sampling time point, 68%, 67% and 49% of all metabolites that showed a significant change in content across all time points and cultivars were significantly altered in N22, Dular and Anjali, respectively. From this comparison between flag leaves and developing seeds we may hypothesize that seeds showed a higher rate of metabolic change than flag leaves. In particular, the massive reduction in the content of many amino acids and organic acids could argue for a rapid conversion from metabolically active pools to a reserve storage. This is in agreement with metabolomic studies in maize [43] and rice [44] that also found a strong reduction in the levels of many primary metabolites during seed development.

Effects of rewatering on the metabolome of drought and heat-stressed plants

In our previous report [26], we evaluated the metabolic responses of rice to severe combined drought and heat stress. Fifty-five stress-responsive metabolites were identified across the three cultivars in flag leaves at the flowering stage, 51 in flag leaves at the early grain-filling stage, 53 in flowering spikelets, and 28 in developing seeds. Here, we highlight changes of these metabolites between stressed plants before and after rewatering. Additionally, we compare metabolite levels after rewatering with levels under fully flooded control conditions to assess to which extent the plants had recovered from stress.

Flag leaves

In flag leaves at both the flowering and early grain-filling stages, PCA revealed that PC1, which explained 34% of the variance in the data, separated the metabolite profiles of samples from control and stressed plants, with the samples taken after rewatering located between these two extremes (Fig. 1A and B). While the metabolite profiles of flag leaves at the flowering stage collected 12 h after rewatering were still close to the stressed samples, profiles obtained 36 h and 60 h after rewatering were more similar to control conditions, indicating partial metabolic recovery (Fig. 1A). Flag leaves from the early grain-filling stage 36 and 60 h after rewatering approached the metabolite composition of control samples collected in parallel to the stress and 12 h rewatering time points (Fig. 1B), also indicating recovery. However, the data also suggest that the unstressed leaves developed faster, while the drought and heat-stressed leaves suffered a delay in development and did not reach the metabolic composition of the control samples taken 60 h after rewatering. Moreover, the drought-susceptible cultivar Anjali was separated from the

drought-tolerant cultivars N22 and Dular by PC2, which accounted for approximately 24-26% of the total variance.

The metabolic response of flag leaves at the flowering stage to rewatering involved mainly the reduction of metabolite levels in comparison with levels under severe stress (Fig. 4). Twelve h after rewatering, only 10 of the 55 stress-responsive metabolites showed significant differences in their relative levels (Fig. 4A and B). Among seven metabolites with reduced and three with increased levels, eight showed cultivar-specific responses. After 36 h and 60 h of rewatering, approximately 60% of the stress-responsive metabolites showed significant differences compared to their levels under severe stress (Fig. 4C to F). The majority of these metabolites had lower levels during recovery than under stress and more than half of the metabolites with reduced levels 60 h after rewatering were common to either all or any two of the cultivars (Fig. 4F). In spite of these changes during recovery, around 60% of all analyzed metabolites were still significantly different from their control levels at the different rewatering time points (Additional file 1), indicating incomplete metabolic recovery.

Metabolites that showed increased levels in flowering stage flag leaves after rewatering included primarily organic acids such as citric, isocitric and glyceric acid (Fig. 4G). These metabolites exhibited reduced levels under severe drought and heat stress, but in spite of the accumulation during recovery were still significantly lower in some cultivars compared with the well-watered control samples (Additional file 2). On the other hand, several metabolites that increased under severe stress were reduced in all cultivars after rewatering, including raffinose, Glc, Pro, Gly, and N-carboxyglycine (Fig. 4G). Pro was strongly reduced in both drought-tolerant cultivars (N22 and Dular), but showed an earlier response in the combined drought and

heat-tolerant N22. The levels of these metabolites mainly returned to control levels after 60 h of rewatering (Additional file 2).

In flag leaves at the early grain-filling stage, the responses after rewatering were generally similar to those observed at the flowering stage, with an increasing number of metabolites with significantly different levels between rewatering and severe stress over time. The majority of these changes were reductions in metabolite levels (Fig. 5). The responses 12 h after rewatering were mostly cultivar specific, with the drought and heat-susceptible cultivar Anjali showing the highest number of significant changes (Fig. 5A and B). N22 and Anjali had also accumulated some common metabolites 36 h after rewatering, but none of them were in common with Dular (Fig. 5C). In contrast, among the metabolites that exhibited reduced levels after rewatering, more than one-third (9 metabolites) were common among all three cultivars at 36 h after rewatering (Fig. 5D), out of which six were amino acids (Fig. 5G). N22 showed a more similar response with the equally drought-tolerant Dular 60 h after rewatering than with the susceptible cultivar Anjali, while there was no exclusive overlap between the heat-susceptible cultivars Dular and Anjali (Fig. 5E and F). Moreover, metabolic recovery was again only partial, with up to 59% of all analyzed metabolites having significantly different levels between samples from rewatered compared to the corresponding well-watered plants (Additional file 3).

Similar to the response of flag leaves at the flowering stage, the levels of the TCA cycle intermediates isocitric and citric acid also increased after rewatering in flag leaves at the early grain-filling stage, accompanied by a decrease in amino acid and sugar levels (Fig. 5G). In the case of isocitric acid, the response was exhibited only by N22 and Anjali (Fig. 5G), in contrast to a general response of all cultivars in flag leaves during flowering (Fig. 4G). Even with this increase in levels after rewatering from reduced levels during severe stress, the magnitude of

change was not sufficient to reach control levels (Additional file 4). Conversely, all cultivars showed reduced levels of several metabolites, including Ile, Val, raffinose, 2-oxo-glutaric acid, and Pro in flag leaves during early grain filling, with a cultivar-dependent extent and timing of change (Fig. 5G). While Pro levels quickly returned to control values during rewatering, raffinose levels remained higher than the constitutive levels (Additional file 4), similar to the observation in flag leaves at the flowering stage. Interestingly, the levels of Suc, a well-known compatible solute that is frequently found accumulated in plants under various stress conditions, were not increased under severe stress in flag leaves at either developmental stage and also did not consistently change after rewatering.

The only directly comparable metabolomic data obtained using a similar experimental design was generated from eucalypts, where the accumulation of metabolites, mostly amino acids, during combined drought and heat stress was reversed during recovery [39], which we have also observed for most of the stress-induced metabolites. In switchgrass exposed to drought stress and rewatering, the metabolite profile after 4 h of recovery was not significantly different from that of the stress condition [45], which is in line with our finding that plants under stress and 12 h after rewatering have similar metabolic profiles. Meanwhile, the metabolome of Arabidopsis leaves has been investigated after cold acclimation at 4 °C and after a subsequent shift back to control temperatures (20 °C). Quite strikingly, in this study a similar strong reduction of the levels of metabolites that were accumulated in the cold was observed [45]. This reversal of the metabolic stress effect, however, was even stronger and more rapid (within 24 h) in Arabidopsis after the temperature shift than it was in rice after rewatering. This may in part be due to the fact that a temperature shift can be experimentally performed much more rapidly by immediate transfer of plants between climate chambers than rewatering a rice field. In addition,

and perhaps more importantly, temperature equilibration of Arabidopsis plants after the shift was likely faster than the recovery of water status in rice plants.

Flowering spikelets

In the PCA (Fig. 1C) the metabolite profiles of flowering spikelets were separated between rewatering time points and cultivars. This was similar to flag leaves, but the distinction between cultivars contributed more (31%) to the overall variance than the separation between time points (21%). In addition, the control, stress and 12 h rewatering samples clustered much more closely together in N22 than in Dular and Anjali.

In total, 77% of the 53 stress-responsive metabolites in flowering spikelets differed significantly in at least one cultivar during at least one of the rewatering time points compared to the severe drought and heat treatment. Similar to what we described for flag leaves above, most of these metabolites showed significantly reduced levels (Fig. 6). Twelve h after rewatering, 24 metabolites showed a decrease in Dular and/or Anjali, while only four metabolites responded in N22 (Fig. 6B). N22 had two common metabolic responses each with one of the other cultivars, while Dular and Anjali had nine metabolites in common, of which most were amino acids (Fig. 6G). Conversely, the six metabolites that showed increased levels 12 h after rewatering were all cultivar specific (Fig. 6A) and the same was true for the two metabolites with increased levels 60 h after rewatering (Fig. 6 E). At 36 and 60 h after rewatering, the number of metabolites with significantly changed levels that were common between all three cultivars increased to eight (Fig. 6D) and nine (Fig. 6F), respectively. In addition to ribitol, which was already common between all cultivars 12 h after rewatering, seven amino acids exhibited lower levels relative to the stressed condition after 36 h. After 60 h the unidentified metabolite A170001 was in addition

commonly reduced in all cultivars. Five of these amino acids, namely Gly, Ile, Leu, Tyr, and Val, already had reduced levels 12 h after rewatering in the combined drought and heat-susceptible cultivars Dular and Anjali (Fig. 6G). Nevertheless, only about 30% of the 88 metabolites analyzed in flowering spikelets had reverted back to control levels after rewatering (Additional file 5), indicating incomplete metabolic recovery also in flowering spikelets (Additional file 6).

Developing seeds

In the PCA of the metabolite profiles of developing seeds (Fig. 1D), PC1 (43% of the total variance) separated the developing seed samples according to the time after rewatering, while PC2 (25% of the total variance) separated metabolite profiles of Dular from N22 and Anjali. Interestingly, there was no clear separation between samples from plants that were grown under control conditions at the different time points and samples from plants that had experienced severe drought and heat stress and rewatering.

Among the investigated organs, developing seeds had shown the smallest number of metabolites with significantly altered levels under severe stress conditions [26]. Consequently, the number of metabolites that were significantly influenced by rewatering compared to the stressed state was also quite low (Fig. 7). We only observed one, four and two metabolites that showed an increase 12 h, 36 h, and 60 h after rewatering (Fig. 7A, C, E) and only raffinose content in Anjali was increased at all time points (Fig. 7G). However, it was also increased in N22 and Dular at the later time points. This typical stress-induced osmolyte was specifically further accumulated after rewatering in developing seeds, while it was massively reduced after

rewatering in flag leaves. However, this may be a developmental effect, as raffinose accumulates in rice during seed development, independent of stress effects [46].

The number of metabolites in developing seeds that showed significantly lower levels than under stress increased over time after rewatering from six to 14 and 20 after 12 h, 36 h and 60 h (Fig. 7B, D, F, G), similar to the response of flag leaves and flowering spikelets. This corresponded to a time-dependent decrease in the number of metabolites whose levels were significantly different from constitutive levels (Additional file 7). The decrease in the levels of Ile and Thr in developing seeds 12 h after rewatering relative to the levels under stress resulted in relative concentrations similar to those under control conditions in all cultivars at this time point (Additional file 8). The further reduction after 36 h led to significantly lower Ile and Thr content than in the control samples in N22 and Anjali. However, these levels increased again and approached the control values 60 h after rewatering.

Correlations between metabolite composition after rewatering and grain yield and quality

We have previously identified potential marker metabolites for tolerance to combined drought and heat stress expressed as the stability of grain yield and quality under stress [26]. These markers were identified from the metabolomes of the three cultivars under control and severe stress conditions. Here, we identified additional metabolite marker candidates from the metabolomes of the three rice cultivars after rewatering. We tested the correlation between changes in metabolite levels before (severe stress) and after rewatering, and the stress-induced reduction in grain yield and increase in proportion of chalky grains (i.e. percentage of grains with >50% chalk content). To determine the magnitude of metabolic changes we compared metabolite content between stressed plants and plants 60 h after rewatering as an indirect measure of the

speed of metabolic recovery from stress. A positive correlation from this analysis indicates that larger changes in the content of a metabolite during rewatering are associated with either a smaller yield reduction or a larger increase in the fraction of chalky grains.

Correlations between changes in metabolite levels after rewatering and yield reduction under stress

The correlation analysis between changes in metabolite levels after rewatering and the drought and heat stress-induced reduction in yield identified 28 metabolites with significant correlations (Table 1). Most of the metabolites exhibited a positive correlation, indicating that these metabolites had larger changes in levels after rewatering when stress-induced yield loss was smaller. On the other hand, nine metabolites yielded negative correlations, of which all except A180002 were observed in flag leaves collected during the early grain-filling stage and in developing seeds. Only erythronic acid and the unknown A147011 showed significant correlations for both sink organs (flowering spikelets and developing seeds), while there were no metabolites that showed significant correlations in flag leaves at both developmental stages. Isocitric acid was common between flag leaves at the flowering stage and developing seeds, while phosphoric acid was common in flag leaves at the early grain-filling stage and flowering spikelets In both cases the metabolites showed opposite directions of the correlations in the source and sink organs. Aside from these, all other metabolites were unique to a specific organ at a specific developmental stage. The highest number of significant correlations was detected for metabolites in developing seeds (15 metabolites) and the lowest in flag leaves at the flowering stage (3 metabolites). Flag leaves at the early grain-filling stage and flowering spikelets each had seven metabolites with significant correlations.

Of these 28 metabolites, 17 were also identified in our previous analysis, where we used metabolite changes under stress and constitutive metabolite content for correlation with yield reduction [26]. Interestingly, there were six metabolites that were identified in the same organ in both studies: glycerophosphoglycerol in flag leaves at the flowering stage from the change in level under severe stress and after rewatering; dehydroascorbic acid dimer in flag leaves at the early grain-filling stage from the constitutive metabolite levels and the rewatering response; malic acid in flag leaves at the early grain-filling stage under severe stress and after rewatering; erythronic acid in flowering spikelets under severe stress and after rewatering; isocitric acid in developing seeds under severe stress and after rewatering; and pyruvic acid in developing seeds under severe stress and after rewatering. In addition, erythronic and threonic acid were each identified in a total of five different organs/treatments, and isocitric, phosphoric and gluconic acid in four different organs/treatments. We hypothesize that these metabolites are particularly promising candidates as markers to select for yield stability under combined drought and heat stress. Obviously, this hypothesis needs further testing with a larger panel of genotypes.

Correlations between changes in metabolite levels after rewatering and the increase in the proportion of chalky grains under stress

Only six metabolites showed significant correlations between their changes 60 h after rewatering and the increase in the proportion of chalky grains under stress (Table 2). Five of these metabolites showed significant correlations in flag leaves, and one in developing seeds. Four of the metabolites identified in flag leaves showed negative correlations (glycerophosphoglycerol, sucrose, A137012, A170001), indicating that larger changes after rewatering were associated with smaller increases in the fraction of chalky grains, i.e. higher

tolerance to combined drought and heat stress. The other two metabolites showed positive correlations either in flag leaves (*trans*-sinapic acid) or in developing seeds (arabitol). Only arabitol showed an overlap with the marker metabolite candidates for seed quality stability under combined drought and heat stress that were identified in our previous investigation, however, in a different organ and treatment [26].

Table 1. Correlation between yield reduction and changes in metabolite levels. Metabolites with significant correlations (Spearman's rank correlation, $P < 0.05$) between stress-induced yield reduction and the changes in metabolite levels (expressed as $\log_2$-fold change) 60 h after rewatering relative to severe stress are shown together with the corresponding correlation coefficients. The analysis was performed for metabolites from flag leaves collected during the flowering and early grain-filling stages, flowering spikelets and developing seeds. Metabolites in bold font are common between the two sink organs. Metabolites are sorted alphabetically.

Organ/ Developmental stage	Metabolite	Correlation coefficient
Flag leaves	Glycerophosphoglycerol	0.72
Flowering stage	Isocitric acid	0.77
	Ribitol	0.72
Flag leaves	A116014	0.80
Early grain-filling stage	A214004	0.83
	Dehydroascorbic acid dimer	0.87
	Glyceric acid	-0.75
	Glycine	-0.88
	Malic acid	-0.83
	Phosphoric acid	-0.78
Flowering spikelets	**A147011**	**0.73**
	A180002	-0.78
	Arbutin	0.82
	Aspartic acid	0.72
	**Erythronic acid**	**0.73**
	Galactonic acid	0.73
	Phosphoric acid	0.77
Developing seeds	**A147011**	**0.77**

	A203003	0.73
	A311002	0.72
	**Erythronic acid**	**0.70**
	Fructose	0.77
	Gluconic acid	0.70
	Glucose	0.77
	myo-Inositol-phosphate	0.70
	Isocitric acid	-0.72
	Kestose, 1-	0.80
	N-Carboxyglycine	0.83
	Pyridine, 2-hydroxy-	-0.75
	Pyridine, 3-hydroxy-	-0.73
	Pyruvic acid	-0.92
	Threonic acid	0.73

Table 2. Correlation between increase in the fraction of chalky grains and changes in metabolite levels. Metabolites with significant correlations (Spearman's rank correlation, $P < 0.05$) between stress-induced increase in the proportion of chalky grains and the changes in metabolite levels (expressed as $\log_2$-fold change) 60 h after rewatering relative to severe stress are shown together with the corresponding correlation coefficients. The analysis was performed for metabolites from flag leaves collected during the flowering and early grain-filling stages, flowering spikelets and developing seeds. However, no metabolite showed significant correlation in flowering spikelets. Metabolites are sorted alphabetically.

**Organ/ Developmental stage**	**Metabolite**	**Correlation coefficient**
Flag leaves	Glycerophosphoglycerol	-0.80
Flowering stage	*trans*-Sinapic acid	0.78
	Sucrose	-0.70
Flag leaves	A137012	-0.70
Early grain-filling stage	A170001	-0.75
Developing seeds	Arabitol	0.78

Potential implications

Our analysis showed that under well-watered control conditions significant metabolic changes occurred over a period of only three days. These changes were particularly pronounced in developing seeds, while the metabolome of flag leaves was much more stable. This implies that the choice of reference point to determine metabolic changes due to different treatments can significantly influence the final results and interpretations, and that the effect of this choice will even depend on the investigated organ. Since development, and therefore changes in metabolites that are unrelated to stress effects, may occur at a different rate under stressed compared to non-stressed conditions, it may be virtually impossible to determine a single "absolutely correct" time point to use as the control. We suggest that the only solution to this problem is a cautious and very careful interpretation of such data, taking into account developmental changes in metabolite levels of the organ of interest.

Secondly, our data suggest that while many stress-responsive metabolites returned to (almost) control levels within three days after stress relief, this was clearly not true for all such metabolites. While this may in part be due to the additional developmental effects on metabolites as discussed above, these persistent metabolic changes are a sign of metabolic imprinting [48]. Metabolic imprints may lead to a modified stress response under a recurrent stress situation. Such phenomena have been defined in the recent literature as stress memory (see [49] for a review). While the current study did not investigate stress memory effects, this aspect clearly warrants further research, also in the light of the predicted increase in erratic weather patterns due to global climate change.

Methods

Experimental setup

Crop husbandry and treatment imposition were performed as described in our previous report [25]. Three rice (*Oryza sativa* L.) cultivars were grown in the field at the International Rice Research Institute (IRRI), Philippines during the dry seasons of three consecutive years (2013 – 2015). The cultivars N22 (*aus* ssp.; drought, heat and combined drought and heat tolerant), Dular (*aus* ssp.; drought tolerant, heat and combined drought and heat susceptible), and Anjali (*indica* ssp.; drought, heat, and combined drought and heat susceptible), which were selected based on their differential responses to independent or combined drought and heat stress during the reproductive stage [23,50–52] were used in these experiments. Plants were staggered-sown in separate plots allocated for drought imposition during flowering and early grain filling. This planting approach allowed for the two developmental stages to occur simultaneously in the three cultivars during late April to early May, which is the hottest period at IRRI. Consequently, the three-day rewatering period that we monitored occurred in early- to mid-May, which recorded an average maximum ambient air temperature of 33.8 ± 0.83 °C across the three years, compared with 34.3 ± 0.50 °C during the stress period. On the last day of the drought stress treatment, when the soil water potential had reached an average of -46.6 ± 11.1 kPa across all experiments [25], the drought-stressed plots were rewatered starting at 18:00. It took ~3 h until the plots were fully irrigated and they were subsequently kept fully flooded until harvest. In parallel, control plots were kept fully flooded throughout the experiment. It should be pointed out that a true control, i.e. growth under well-watered conditions with lower air temperatures, was not possible to include in this type of field experiment.

Sample collection

Flag leaves, flowering spikelets, and developing seeds were collected in three to five replicates per cultivar from control plots, at the end of the drought stress period and during the first three days of rewatering. We collected 385 samples in 2013, 376 in 2014 and 390 in 2015, making up a total of 1151 samples that were analysed by GC-MS. Combining replicates from the three years, we obtained 15 replicates in approximately 83% and 14 replicates in approximately 10% of all cases, i.e. tissues, cultivars and treatments. The sampling time was between 9:00 – 11:30 to avoid the effects of circadian rhythms on metabolite content. Since the plots were fully flooded at ~21:00 on the day of irrigation after the drought treatment, the collection of samples during the first rewatering time point (i.e. on the following day) corresponded to 12 h of rewatering. The subsequent time points, which were on consecutive days, were thus at 36 h and 60 h after rewatering.

Spikelets flowering at the time of sampling, as well as flag leaves at that developmental stage, were collected from the flowering stage drought-stress plots. The samples from stressed plants were the same as those denoted “severe stress” in our previous report [26]. It should be noted that most of the spikelets were from panicles trapped within the flag leaf sheath during the drought stress treatment and were just exserted upon rewatering. The corresponding control samples for flowering spikelets and flag leaves at the flowering stage were collected only once from the control plot and were the same samples as described previously [26]. Developing seeds, which were marked as flowering spikelets during the first few days of drought stress (see [26] for details), were collected from both the corresponding control and stress plots at every rewatering time point. The developing seeds were collected 10-12 days after flowering (DAF), 11-13 DAF, and 12-14 DAF for the 12 h, 36 h, and 60 h rewatering time points, respectively, across the three

experiments. In addition, flag leaves from tillers with panicles at the grain-filling stage were collected from the control and early grain-filling stage drought-stress plots. Further details of the sample collection have been described previously [26].

Metabolite profiling and data processing

Metabolite profiling and data processing was performed as reported by Lawas et al. [26]. A fraction enriched in small primary and secondary metabolites was extracted from liquid nitrogen quenched ground tissue samples and was analyzed by gas chromatography coupled to electron impact ionization-time of flight-mass spectrometry (GC/EI-TOF-MS) as previously described [53]. The mass spectral intensities of identified metabolites were normalized to sample fresh weight and $^{13}C_6$-sorbitol as internal standard. All metabolomics data are freely available [41, 54]

Statistical analysis

Statistical analyses were executed using R version 3.4.0 [55] and RStudio version 1.0.153 [56]. Data pre-processing (handling of missing values, normalization to remove effects of measurement batch and sequence, outlier detection, normalization and transformation) prior to the main statistical analyses were the same as in our previous report [26], where we emphasized that all data pre-processing was performed including all samples collected during the stress and rewatering time points to enable direct comparisons. Pre-processed data from the three experiments were combined into one data set for each organ per developmental stage (flag leaves at the flowering stage, flag leaves at the early grain-filling stage, flowering spikelets, developing seeds). Mean values of samples collected during the control, stress, and rewatering time points were Pareto-scaled and mean-centered, and subjected to PCA using the probabilistic method

from the *'pcaMethods'* package (version 1.60.0) [57]. Scores obtained from the PCA were plotted using the *'ggplot2'* package (version 2.2.1). Differences between metabolite levels of flag leaves collected during the early grain-filling stage and of developing seeds obtained at different time points under fully flooded control conditions were assessed by comparing the relative metabolite levels (median-normalized and log_2-transformed values) of control samples collected during each of the rewatering time points to the control samples collected in parallel to the stress time point (rewatering time points 0 h to 60 h). In addition, we compared the relative levels of metabolites that were significantly responsive to severe stress [26] before and after rewatering in each of the organs. The relative metabolite levels during rewatering were also compared to the relative metabolite levels under control conditions to evaluate how far the stress effects were reversed. In this case, all metabolites (i.e. not only the stress-responsive metabolites) were included in the analysis. All comparisons were performed using the Wilcoxon-Mann-Whitney test after assessing the normality of the data by the Shapiro-Wilk test (R package *'stats'*, version 3.4.0). Metabolites that showed significant differences in the comparisons were plotted in Venn diagrams (*'VennDiagram'* package, version 1.6.17) and in heat maps with hierarchical clustering using Euclidean distance and average linkage ('*gplots'* package, version 3.0.1). Correlation analysis between the stress-induced changes in grain yield and quality (measured in terms of the proportion of "chalky grains", i.e. grains with >50% chalk content) and in the change in metabolite levels between the 60 h rewatering and the stress time points was performed using the Spearman's rank method (R package *'stats'*). Data on grain yield and quality from our previous report [25] was used. Pre-processed metabolite data was median-normalized and log_2-transformed per experiment. A total of nine values (three cultivars x three years) were used for the correlation tests. All code used in these analyses is freely available [58].

Availability of supporting data and materials

The data set supporting the results of this article is available in the EMBL-EBI MetaboLights database [41] (DOI: 10.1093/nar/gks1004; PubMed PMID: 23109552) with the identifier MTBLS801. Snapshots of our code and other data supporting this research are available in the *GigaScience* repository, GigaDB [42, 59].

Availability of source code and requirements

Project name: Rice_HxD_Recovery_Metabolomics

Project home page: GitHub (https://github.com/llawas/Rice_HxD_Recovery_Metabolomics)

Operating system: Windows 7

Programming language: R

License: GNU General Public License

RRID: SCR_017204

Abbreviations

DAF: days after flowering; GC-MS: gas chromatography-mass spectrometry; Glc: glucose; PCA: principal component analysis; Suc: sucrose; TCA cycle: tricarboxylic acid cycle

Acknowledgements

LMFL gratefully acknowledges support by a PhD fellowship from the University of Potsdam, Germany. We thank Dr. Xia Li (present address: Institute of Subtropical Agriculture, Chinese Academy of Science, Changsha, Hunan, 410125, China) for help with sampling.

Funding

This project has been supported by grants from the German Federal Ministry for Economic Cooperation and Development [Project Number 11.7860.7-001.00; Contract Numbers 81141844 and 81170348] to SVKJ and by the Max-Planck Society to JK and DKH. The funding bodies had no role in study design, data collection, analysis or interpretation, or in writing the manuscript.

Authors' contributions

SVKJ and DKH conceived the project. SVKJ and LMFL organized the field experiments, LMFL performed the sampling. AE and JK performed the metabolomic analysis and metabolite annotation. LMFL performed the data analysis with contributions from EZ and DKH. LMFL and DKH wrote the manuscript with contributions from all co-authors.

Competing interests

The authors declare that they have no financial or non-financial competing interests.

References

1. Lesk C, Rowhani P, Ramankutty N. Influence of extreme weather disasters on global crop production. Nature. 2016;529:84–7.

2. Hatfield JL, Boote KJ, Kimball BA, Ziska LH, Izaurralde RC, Ort D, et al. Climate impacts on agriculture: Implications for crop production. Agron J. 2011;103:351–70.

3. Coumou D, Rahmstorf S. A decade of weather extremes. Nat Clim Chang. 2012;2:491–6.

4. IPCC. Climate Change 2014: Synthesis Report. Contribution of Working Groups I, II and III to the Fifth Assessment Report of the Intergovernmental Panel on Climate Change. Core Writing Team, Pachauri RK, Meyer LA, editors. Geneva, Switzerland: Cambridge University Press; 2014.

5. Dunn RJH, Stanitski DM, Gobron N, Willett KM, editors. Global Climate State 2017. Bulletin of the American Meteorological Society; 2018. p. S5–68.

6. Battisti DS, Naylor RL. Historical warnings of future food insecurity with unprecedented seasonal heat. Science. 2009;323:240–4.

7. Touma D, Ashfaq M, Nayak MA, Kao S-C, Diffenbaugh NS. A multi-model and multi-index evaluation of drought characteristics in the 21st century. J Hydrol. 2015;526:196–207.

8. Lobell DB, Gourdji SM. The influence of climate change on global crop productivity. Plant Physiol. 2012;160:1686–97.

9. Madadgar S, AghaKouchak A, Farahmand A, Davis SJ. Probabilistic estimates of drought impacts on agricultural production. Geophys Res Lett. 2017;44:7799–807.

10. Zhao C, Liu B, Piao S, Wang X, Lobell DB, Huang Y, et al. Temperature increase reduces global yields of major crops in four independent estimates. Proc Natl Acad Sci USA. 2017;114:9326–31.

11. Li Y, Ye W, Wang M, Yan X. Climate change and drought: a risk assessment of crop-yield impacts. Clim Res. 2009;39:31–46.

12. Hasegawa T, Ishimaru T, Kondo M, Kuwagata T, Yoshimoto M, Fukuoka M. Spikelet sterility of rice observed in the record hot summer of 2007 and the factors associated with its variation. J Agric Meteorol. 2011;67:225–32.

13. Pandey S, Bhandari H, Ding S, Prapertchob P, Sharan R, Naik D, et al. Coping with drought in rice farming in Asia: Insights from a cross-country comparative study. Agric Econ. 2007;37:213–24.

14. GRiSP (Global Rice Science Partnership). Rice almanac. 4th ed. Los Baños (Philippines): International Rice Research Institute; 2013.

15. Jagadish SVK, Muthurajan R, Oane R, Wheeler TR, Heuer S, Bennett J, et al. Physiological and proteomic approaches to address heat tolerance during anthesis in rice (*Oryza sativa* L.). J Exp Bot. 2010;61:143–56.

16. Shi W, Lawas LMF, Raju BR, Jagadish SVK. Acquired thermo-tolerance and trans-generational heat stress response at flowering in rice. J Agron Crop Sci. 2016;202:309–19.

17. Shi W, Li X, Schmidt RC, Struik PC, Yin X, Jagadish SVK. Pollen germination and in vivo fertilization in response to high-temperature during flowering in hybrid and inbred rice. Plant Cell Environ. 2018;41:1287–97.

18. Suriyasak C, Harano K, Tanamachi K, Matsuo K, Tamada A, Iwaya-Inoue M, et al. Reactive oxygen species induced by heat stress during grain filling of rice (*Oryza sativa* L.) are involved in occurrence of grain chalkiness. J Plant Physiol. 2017;216:52–7.

19. Kadam NN, Struik PC, Rebolledo MC, Yin X, Jagadish SVK. Genome-wide association reveals novel genomic loci controlling rice grain yield and its component traits under water-deficit stress during the reproductive stage. J Exp Bot. 2018;69:4017–32.

20. Gowda VRP, Henry A, Vadez V, Shashidhar HE, Serraj R. Water uptake dynamics under progressive drought stress in diverse accessions of the OryzaSNP panel of rice (*Oryza sativa*). Funct Plant Biol. 2012;39:402–11.

21. Pantuwan G, Fukai S, Cooper M, Rajataserreekul S, O'Toole JC. Yield response of rice (*Oryza sativa* L.) genotypes to different types of drought under rainfed lowlands. Part 1. Grain yield and yield components. Field Crop Res. 2002;73:153–68.

22. Jagadish SVK, Muthurajan R, Rang ZW, Malo R, Heuer S, Bennett J, et al. Spikelet proteomic response to combined water deficit and heat stress in rice (*Oryza sativa* cv. N22). Rice. 2011;4:1–11.

23. Rang ZW, Jagadish SVK, Zhou QM, Craufurd PQ, Heuer S. Effect of high temperature and water stress on pollen germination and spikelet fertility in rice. Environ Exp Bot. 2011;70:58–65.

24. Li X, Lawas LMF, Malo R, Glaubitz U, Erban A, Mauleon R, et al. Metabolic and transcriptomic signatures of rice floral organs reveal sugar starvation as a factor in reproductive failure under heat and drought stress. Plant Cell Environ. 2015;38:2171–92.

25. Lawas LMF, Shi W, Yoshimoto M, Hasegawa T, Hincha DK, Zuther E, et al. Combined drought and heat stress impact during flowering and grain filling in contrasting rice cultivars grown under field conditions. Field Crop Res. 2018;229:66–77.

26. Lawas LMF, Erban A, Kopka J, Jagadish SVK, Zuther E, Hincha DK. Metabolic responses of rice cultivars with different tolerance to combined drought and heat stress under field conditions. GigaScience. 2019;8:1-21 (DOI: 10.1093/gigascience/giz050).

27. Mahalingam R. Consideration of Combined Stress: A Crucial Paradigm for Improving Multiple Stress Tolerance in Plants. In: Mahalingam R, editor. Combined Stress in Plants: Physiological, Molecular and Biochemical Aspects. Cham: Springer International Publishing; 2015. p. 1–25.

28. Lawas LMF, Zuther E, Jagadish SVK, Hincha DK. Molecular mechanisms of combined heat and drought stress resilience in cereals. Curr Opin Plant Biol. 2018;45:212–7.

29. Mittler R. Abiotic stress, the field environment and stress combination. Trends Plant Sci. 2006;11:15–9.

30. Crisp PA, Ganguly D, Eichten SR, Borevitz JO, Pogson BJ. Reconsidering plant memory: Intersections between stress recovery, RNA turnover, and epigenetics. Sci Adv. 2016;2:e1501340.

31. Yeung E, van Veen H, Vashisht D, Sobral Paiva AL, Hummel M, Rankenberg T, et al. A stress recovery signaling network for enhanced flooding tolerance in *Arabidopsis thaliana*. Proc Natl Acad Sci USA. 2018;115:E6085–94.

32. Chen D, Wang S, Cao B, Cao D, Leng G, Li H, et al. Genotypic variation in growth and physiological response to drought stress and re-watering reveals the critical role of recovery in drought adaptation in maize seedlings. Front Plant Sci. 2016;6:1241.

33. Xu Z, Zhou G, Shimizu H. Plant responses to drought and rewatering. Plant Signal Behav. 2010;5:649–54.

34. Sailaja B, Anjum N, Vishnu Prasanth V, Sarla N, Subrahmanyam D, Voleti SR, et al. Comparative study of susceptible and tolerant genotype reveals efficient recovery and root system contributes to heat stress tolerance in rice. Plant Mol Biol Report. 2014;32:1228–40.

35. Sarkar NK, Kim YK, Grover A. Coexpression network analysis associated with call of rice seedlings for encountering heat stress. Plant Mol Biol. 2014;84:125–43.

36. Muthurajan R, Shobbar ZS, Jagadish SVK, Bruskiewich R, Ismail A, Leung H, et al. Physiological and proteomic responses of rice peduncles to drought stress. Mol Biotechnol. 2011;48:173–82.

37. Liu J-X, Bennett J. Reversible and irreversible drought-induced changes in the anther proteome of rice (*Oryza sativa* L.) genotypes IR64 and Moroberekan. Mol Plant. 2011;4:59–69.

38. Salekdeh GH, Siopongco J, Wade LJ, Ghareyazie B, Bennett J. Proteomic analysis of rice leaves during drought stress and recovery. Proteomics. 2002;2:1131–45.

39. Correia B, Hancock RD, Amaral J, Gomez-Cadenas A, Valledor L, Pinto G. Combined drought and heat activates protective responses in *Eucalyptus globulus* that are not activated when subjected to drought or heat stress alone. Front Plant Sci. 2018;9:819.

40. Zhou R, Kong L, Wu Z, Rosenqvist E, Wang Y, Zhao L, et al. Physiological response of tomatoes at drought, heat and their combination followed by recovery. Physiol Plant. 2019;165:144-54.

41. MetaboLights Database. https://www.ebi.ac.uk/metabolights/. Accessed 14 March 2019.

42. Lawas LMF, Li X, Erban A, Kopka J, Jagadish SVK, Zuther E, et al. Supporting data for "Metabolic responses of rice cultivars with different tolerance to combined drought and heat stress under field conditions." GigaScience Database. 2019; http://dx.doi.org/10.5524/100582.

43. Hu C, Li Q, Shen X, Quan S, Lin H, Duan L, et al. Characterization of factors underlying the metabolic shifts in developing kernels of colored maize. Sci Rep. 2016;6:35479.

44. Hu C, Tohge T, Chan SA, Song Y, Rao J, Cui B, et al. Identification of conserved and diverse metabolic shifts during rice grain development. Sci Rep. 2016;6:20942.

45. Meyer E, Aspinwall MJ, Lowry DB, Palacio-Mejía JD, Logan TL, Fay PA, et al. Integrating transcriptional, metabolomic, and physiological responses to drought stress and recovery in switchgrass (*Panicum virgatum* L.). BMC Genomics. 2014;15:527.

46. Pagter M, Alpers J, Erban A, Kopka J, Zuther E, Hincha DK. Rapid transcriptional and metabolic regulation of the deacclimation process in cold acclimated *Arabidopsis thaliana*. BMC Genomics. 2017;18:731.

47. Yan S, Huang W, Gao J, Fu H, Liu J. Comparative metabolomic analysis of seed metabolites associated with seed storability in rice (*Oryza sativa* L.) during natural aging. Plant Physiol Biochem. 2018;127:590–8.

48. Schwachtje J, Whitcomb SJ, Firmino AAP, Zuther E, Hincha DK, Kopka J. Induced, imprinted, and primed responses to changing environments: Does metabolism store and process information? Front Plant Sci. 2019;10:106.

49. Hilker M, Schwachtje J, Baier M, Balazadeh S, Bäurle I, Geiselhardt S, et al. Priming and memory of stress responses in organisms lacking a nervous system. Biol Rev. 2016;91:1118–33.

50. Henry A, Gowda VRP, Torres RO, McNally KL, Serraj R. Variation in root system architecture and drought response in rice (*Oryza sativa*): Phenotyping of the OryzaSNP panel in rainfed lowland fields. Field Crop Res. 2011;120:205–14.

51. Kumar A, Dixit S, Ram T, Yadaw RB, Mishra KK, Mandal NP. Breeding high-yielding drought-tolerant rice: genetic variations and conventional and molecular approaches. J Exp Bot. 2014;65:6265–78.

52. Tenorio FA, Ye C, Redoña E, Sierra S, Laza M, Argayoso MA. Screening rice genetic resources for heat tolerance. SABRAO J Breed Genet. 2013;45:371–81.

53. Erban A, Schauer N, Fernie AR, Kopka J. Nonsupervised Construction and Application of Mass Spectral and Retention Time Index Libraries From Time-of-Flight Gas Chromatography-Mass Spectrometry Metabolite Profiles. In: Weckwerth W, editor. Meth Mol Biol. Totowa, NJ: Humana Press Inc.; 2007. p. 19–39.

54. Haug K, Salek RM, Conesa P, et al. MetaboLights-an open-access general purpose repository for metabolomics studies and associated meta-data. Nucleic Acids Res 2013;41(Database issue):D781-6.

55. R Core Team. R: A language and environment for statistical computing. Vienna, Austria: R Foundation for Statistical Computing; 2017. https://www.r-project.org/. Accessed 14 March 2019.

55. RStudio Team. RStudio: Integrated Development Environment for R. Boston, MA: RStudio, Inc.; 2016. https://www.rstudio.com/. Accessed 14 March 2019.

57. Stacklies W, Redestig H, Scholz M, Walther D, Selbig J. pcaMethods - A bioconductor package providing PCA methods for incomplete data. Bioinformatics. 2007;23:1164–7.

58. Rice_HxD_Recovery_Metabolomics Project page. https://github.com/llawas/Rice_HxD_Recovery_Metabolomics_Recovery.

59. Lawas LMF; Erban A; Kopka J; Jagadish SVK; Zuther E; Hincha DK: Supporting data for "Metabolic responses of rice source and sink organs during recovery from combined drought and heat stress in the field". GigaScience Database. 2019. http://dx.doi.org/10.5524/100632.

Figure legends

Figure 1. Principal component analysis (PCA) of rice metabolite profiles

Scores of the first two principal components (PC1 and PC2) from PCA of the metabolite profiles of flag leaves at the flowering stage (A), flag leaves at the early grain-filling stage (B), flowering spikelets (C), and developing seeds (D) collected under control and severe stress conditions, and 12, 36, and 60 h after rewatering. Samples were collected from the cultivars N22, Dular, and Anjali in three experiments (n = 12 – 15 per organ per condition). Scores shown are averages of the median-normalized and log_{10}-transformed values of 81, 88, and 67 metabolites in flag leaves,

flowering spikelets, and developing seeds, respectively, that were detected in common across the three experiments.

Figure 2. Constitutive levels of metabolites in flag leaves during the early grain-filling stage

Flag leaves were collected from three rice cultivars under well-watered control conditions in parallel to collection of samples from plants exposed to severe stress and 12, 36, and 60 h after subsequent rewatering (RW). Metabolites that showed significant (Mann-Whitney-Wilcoxon test, $P < 0.05$) differences in constitutive levels between the control samples at the stress time point and any of the control samples taken at the different time points after rewatering are shown in the heat map. Values are averages of the median-normalized and $\log_2$-transformed relative metabolite content as indicated by the color code. Asterisks indicate the level of significance (* $P < 0.05$; ** $P < 0.01$; *** $P < 0.001$). Note that the first column of each cultivar (Control - Severe stress) has no asterisks since it is the reference for comparison with the other columns.

Figure 3. Constitutive levels of metabolites in developing seeds

Developing seeds were collected from three rice cultivars under well-watered control conditions in parallel to collection of samples from plants exposed to severe stress and 12, 36, and 60 h after subsequent rewatering (RW). Metabolites that showed significant (Mann-Whitney-Wilcoxon test, $P < 0.05$) differences in constitutive levels between the control samples at the stress time point and any of the control samples taken at the different time points after rewatering are shown in the heat map. Values are averages of the median-normalized and $\log_2$-transformed relative metabolite content as indicated by the color code. Asterisks indicate the level of significance (* P

< 0.05; ** P < 0.01; *** P < 0.001). Note that the first column of each cultivar (Control - Severe stress) has no asterisks since it is the reference for comparison with the other columns.

Figure 4. Changes in the levels of stress-responsive metabolites in flag leaves at the flowering stage after rewatering

Venn diagrams show the number of common and cultivar-specific metabolites that showed a significant (Mann-Whitney-Wilcoxon test, $P < 0.05$) increase (A, C, E) or decrease (B, D, F) in levels 12 h (A, B), 36 h (C, D), and 60 h (E, F) after rewatering (RW) relative to severe stress conditions. Numbers in parentheses indicate the total number of metabolites with increased/decreased abundance in each cultivar. The corresponding metabolites are shown in the heat map (G). The values, expressed as $\log_2$-fold change between plants after rewatering and plants under severe stress, are indicated by the color code and hierarchically clustered using Euclidean distance and average linkage. Asterisks indicate the level of significance (* $P < 0.05$; ** $P < 0.01$; *** $P < 0.001$).

Figure 5. Changes in the levels of stress-responsive metabolites in flag leaves at the early grain-filling stage after rewatering

Venn diagrams show the number of common and cultivar-specific metabolites that showed a significant (Mann-Whitney-Wilcoxon test, $P < 0.05$) increase (A, C, E) or decrease (B, D, F) in levels 12 h (A, B), 36 h (C, D), and 60 h (E, F) after rewatering (RW) relative to severe stress conditions. Numbers in parentheses indicate the total number of metabolites with increased/decreased abundance in each cultivar. The corresponding metabolites are shown in the heat map (G). The values, expressed as $\log_2$-fold change between plants after rewatering and

plants under severe stress, are indicated by the color code and hierarchically clustered using Euclidean distance and average linkage. Asterisks indicate the level of significance (* P < 0.05; ** P < 0.01; *** P < 0.001).

Figure 6. Changes in the levels of stress-responsive metabolites in flowering spikelets after rewatering

Venn diagrams show the number of common and cultivar-specific metabolites that showed a significant (Mann-Whitney-Wilcoxon test, P < 0.05) increase (A, C, E) or decrease (B, D, F) in levels 12 h (A, B), 36 h (C, D), and 60 h (E, F) after rewatering (RW) relative to severe stress conditions. Numbers in parentheses indicate the total number of metabolites with increased/decreased abundance in each cultivar. The corresponding metabolites are shown in the heat map (G). The values, expressed as $\log_2$-fold change between plants after rewatering and plants under severe stress, are indicated by the color code and hierarchically clustered using Euclidean distance and average linkage. Asterisks indicate the level of significance (* P < 0.05; ** P < 0.01; *** P < 0.001).

Figure 7. Changes in the levels of stress-responsive metabolites in developing seeds after rewatering

Venn diagrams show the number of common and cultivar-specific metabolites that showed a significant (Mann-Whitney-Wilcoxon test, P < 0.05) increase (A, C, E) or decrease (B, D, F) in levels 12 h (A, B), 36 h (C, D), and 60 h (E, F) after rewatering (RW) relative to severe stress conditions. Numbers in parentheses indicate the total number of metabolites with increased/decreased abundance in each cultivar. The corresponding metabolites are shown in the

heat map (G). The values, expressed as log_2-fold change between plants after rewatering and plants under severe stress, are indicated by the color code and hierarchically clustered using Euclidean distance and average linkage. Asterisks indicate the level of significance (* $P < 0.05$; ** $P < 0.01$; *** $P < 0.001$).

Additional files

Additional file 1 (PDF). Venn diagrams showing the number of metabolites in flag leaves at the flowering stage with altered levels after rewatering relative to control levels

Numbers indicate common and cultivar-specific metabolites with a significant (Mann-Whitney-Wilcoxon test, $P < 0.05$) increase (A, C, E) or decrease (B, D, F) in levels 12 h (A, B), 36 h (C, D), and 60 h (E, F) after post-stress rewatering relative to levels in control plants. Numbers in parentheses indicate the total number of metabolites with increased/decreased abundance in each cultivar.

Additional file 2 (PDF). Heat map of metabolites in flag leaves at the flowering stage with altered levels under stress and/or after rewatering relative to control levels

Metabolites showing significant (Mann-Whitney-Wilcoxon test, $P < 0.05$) changes in levels under severe stress and 12 h, 36 h, and 60 h after rewatering (RW) relative to control levels. Metabolites correspond to those illustrated in the Venn diagrams in Additional file 1. The values, expressed as log_2-fold change in the indicated comparisons, are color coded and hierarchically clustered using Euclidean distance and average linkage. Asterisks indicate the level of significance (* $P < 0.05$; ** $P < 0.01$; *** $P < 0.001$). Metabolites in black font are responsive to

either only stress or to both stress and rewatering, while metabolites in red font are responsive only to rewatering.

Additional file 3 (PDF). Venn diagrams showing the number of metabolites in flag leaves at the early grain-filling stage with altered levels after rewatering relative to control levels

Numbers indicate common and cultivar-specific metabolites with a significant (Mann-Whitney-Wilcoxon test, $P < 0.05$) increase (A, C, E) or decrease (B, D, F) in levels 12 h (A, B), 36 h (C, D), and 60 h (E, F) after rewatering relative to levels in control plants. Numbers in parentheses indicate the total number of metabolites with increased/decreased abundance in each cultivar.

Additional file 4 (PDF). Heat map of metabolites in flag leaves at the early grain-filling stage with altered levels under stress and/or after rewatering relative to control levels

Metabolites showing significant (Mann-Whitney-Wilcoxon test, $P < 0.05$) changes in levels under severe stress and 12 h, 36 h, and 60 h after rewatering (RW) relative to control levels. Metabolites correspond to those illustrated in the Venn diagrams in Additional file 3. The values, expressed as $\log_2$-fold change in the indicated comparisons, are color coded and hierarchically clustered using Euclidean distance and average linkage. Asterisks indicate the level of significance (* $P < 0.05$; ** $P < 0.01$; *** $P < 0.001$). Metabolites in black font are responsive to either only stress or to both stress and rewatering, while metabolites in red font are responsive only to rewatering.

Additional file 5 (PDF). Venn diagrams showing the number of metabolites in flowering spikelets with altered levels after rewatering relative to control levels

Numbers indicate common and cultivar-specific metabolites with a significant (Mann-Whitney-Wilcoxon test, $P < 0.05$) increase (A, C, E) or decrease (B, D, F) in levels 12 h (A, B), 36 h (C, D), and 60 h (E, F) after rewatering relative to levels in control plants. Numbers in parentheses indicate the total number of metabolites with increased/decreased abundance in each cultivar.

Additional file 6 (PDF). Heat map of metabolites in flowering spikelets with altered levels under stress and/or after rewatering relative to control levels

Metabolites showing significant (Mann-Whitney-Wilcoxon test, $P < 0.05$) changes in levels under severe stress and 12 h, 36 h, and 60 h after rewatering (RW) relative to control levels. Metabolites correspond to those illustrated in the Venn diagrams in Additional file 5. The values, expressed as $\log_2$-fold change in the indicated comparisons, are color coded and hierarchically clustered using Euclidean distance and average linkage. Asterisks indicate the level of significance (* $P < 0.05$; ** $P < 0.01$; *** $P < 0.001$). Metabolites in black font are responsive to either only stress or to both stress and rewatering, while metabolites in red font are responsive only to rewatering.

Additional file 7 (PDF). Venn diagrams showing the number of metabolites in developing seeds with altered levels after rewatering relative to control levels

Numbers indicate common and cultivar-specific metabolites with a significant (Mann-Whitney-Wilcoxon test, $P < 0.05$) increase (A, C, E) or decrease (B, D, F) in levels 12 h (A, B), 36 h (C, D), and 60 h (E, F) after rewatering relative to levels in control plants. Numbers in parentheses indicate the total number of metabolites with increased/decreased abundance in each cultivar.

Additional file 8 (PDF). Heat map of metabolites in developing seeds with altered levels under stress and after rewatering relative to control levels

Metabolites showing significant (Mann-Whitney-Wilcoxon test, $P < 0.05$) changes in levels under severe stress and 12 h, 36 h, and 60 h after rewatering (RW) relative to control levels. Metabolites correspond to those illustrated in the Venn diagrams in Additional file 7. The values, expressed as $\log_2$-fold change in the indicated comparisons, are color coded and hierarchically clustered using Euclidean distance and average linkage. Asterisks indicate the level of significance (* $P < 0.05$; ** $P < 0.01$; *** $P < 0.001$). Metabolites in black font are responsive to either only stress or to both stress and rewatering, while metabolites in red font are responsive only to rewatering.

Figure 1

Click here to access/download;Figure;Figure 1_HxD_rewatering.pdf

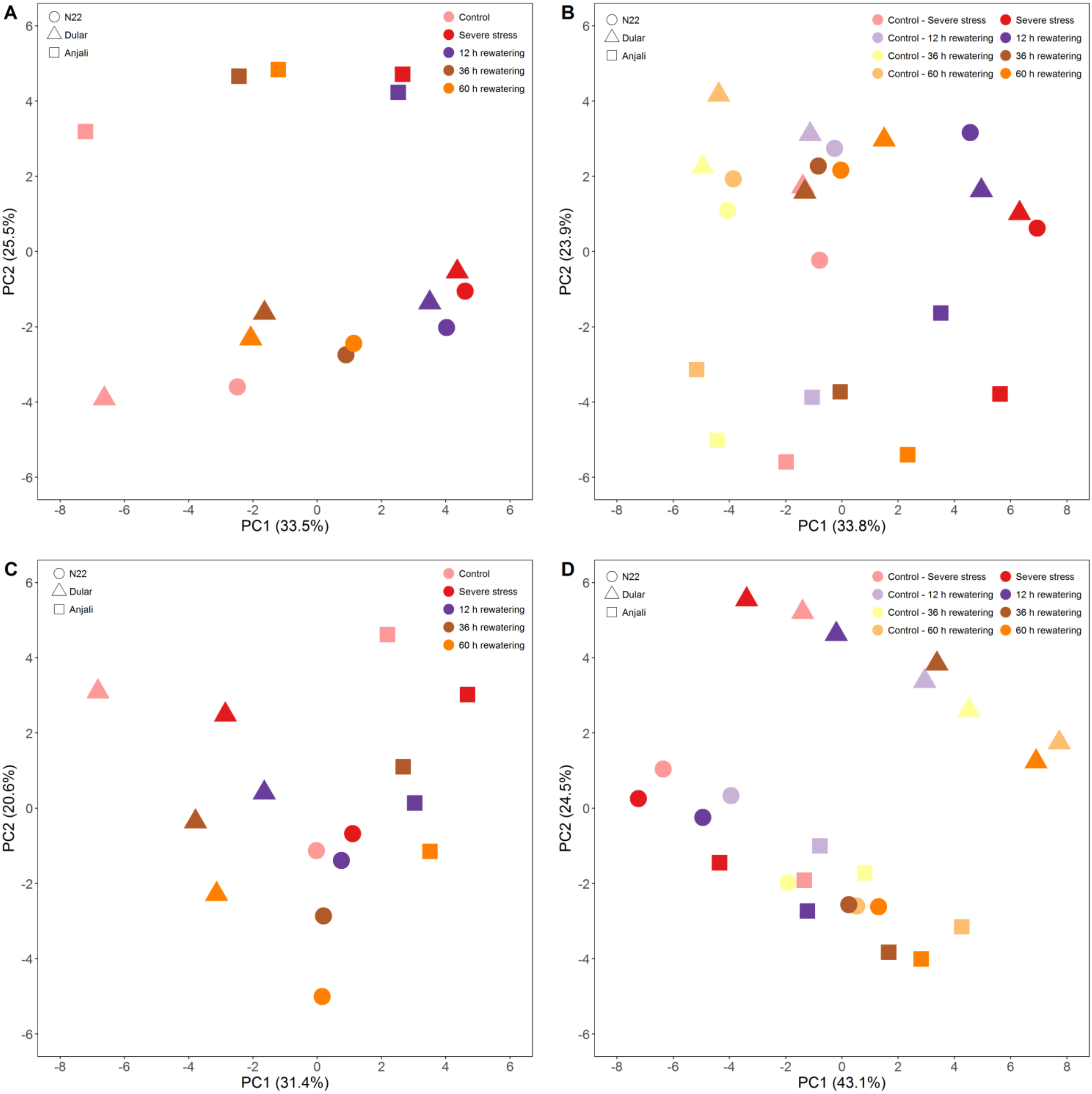

Figure 2

Click here to access/download;Figure;Figure 2_HxD_rewatering.pdf

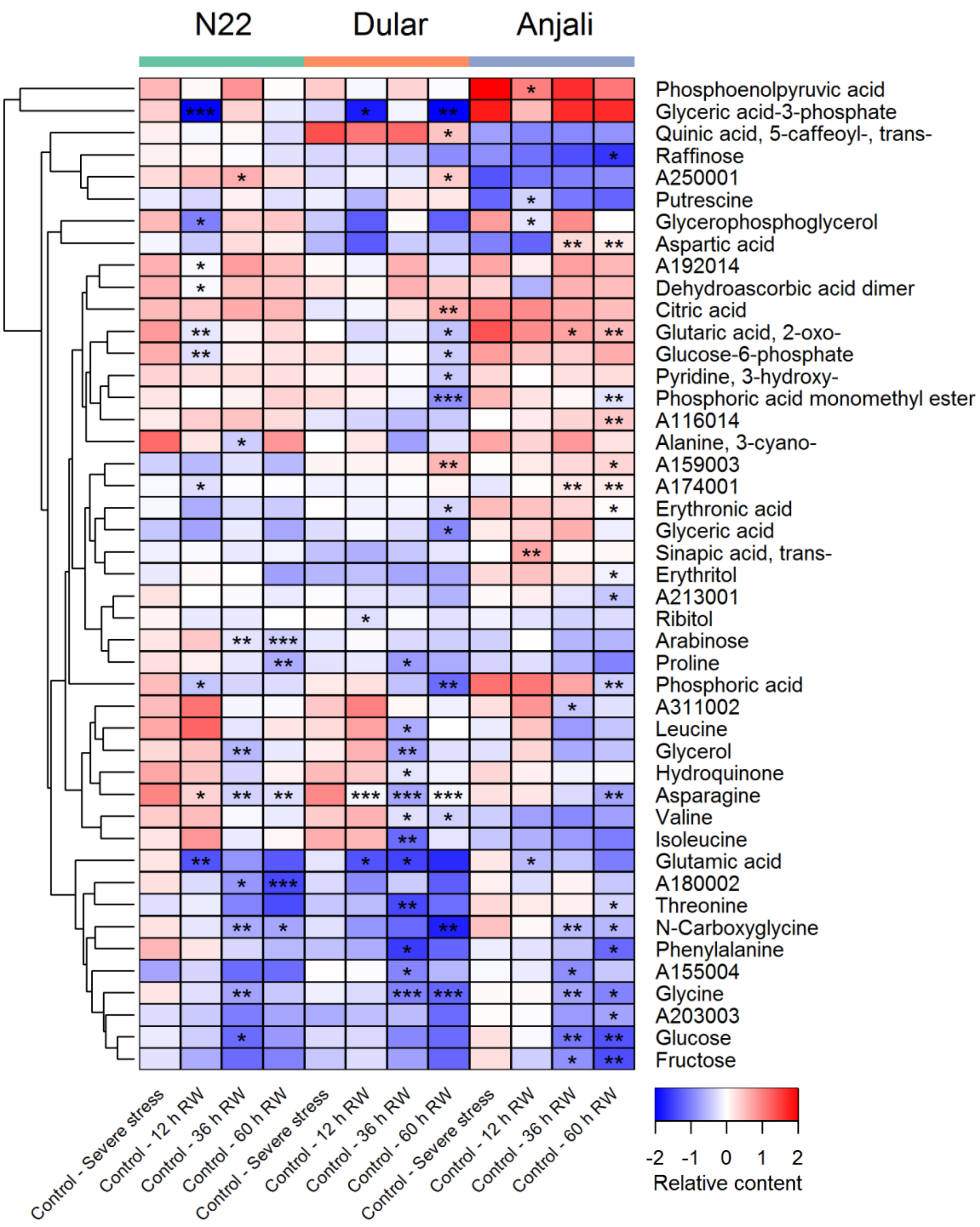

Figure 3

Click here to access/download;Figure;Figure 3_HxD_rewatering.pdf

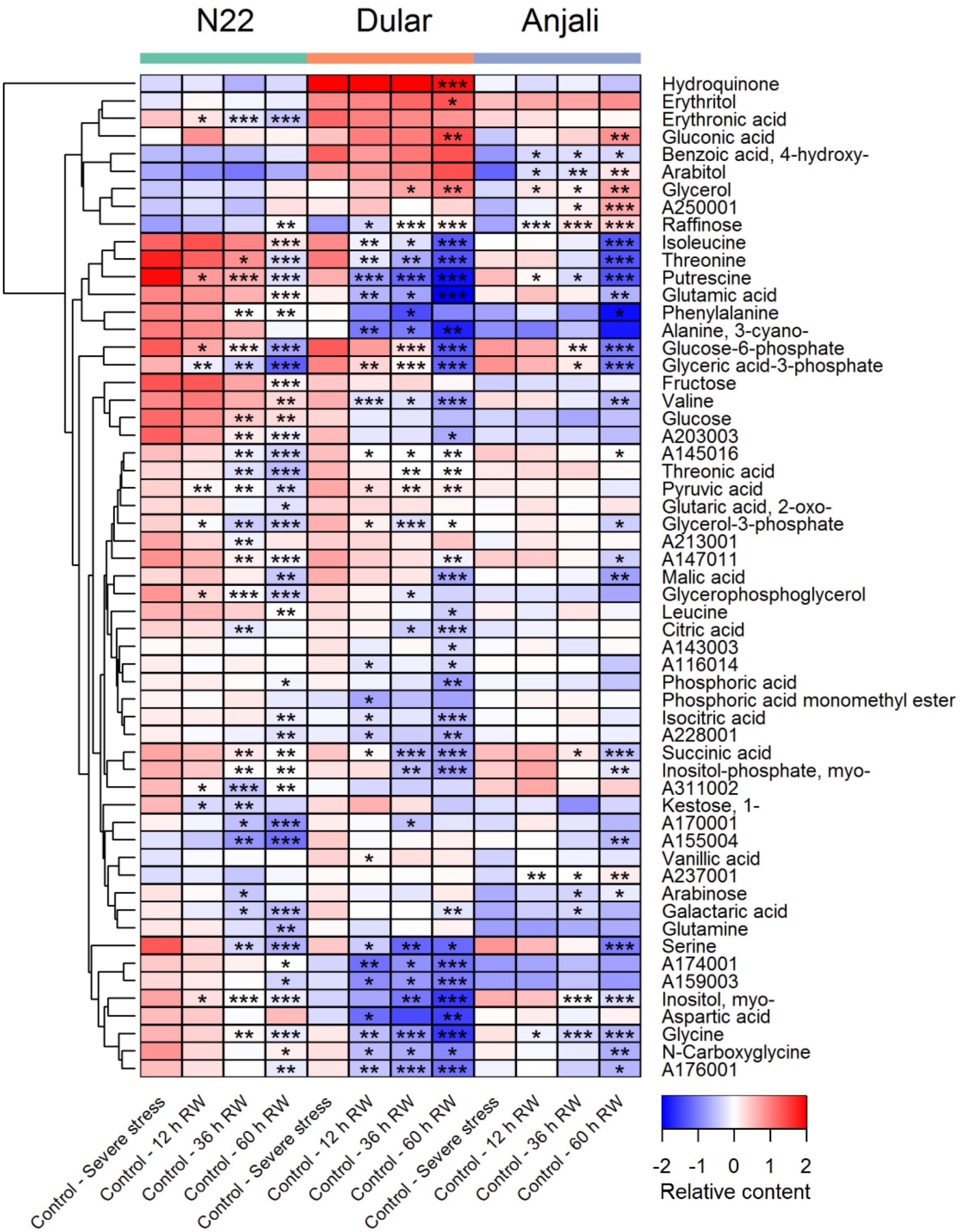

Figure 4

Click here to access/download;Figure;Figure 4_HxD_rewatering.pdf

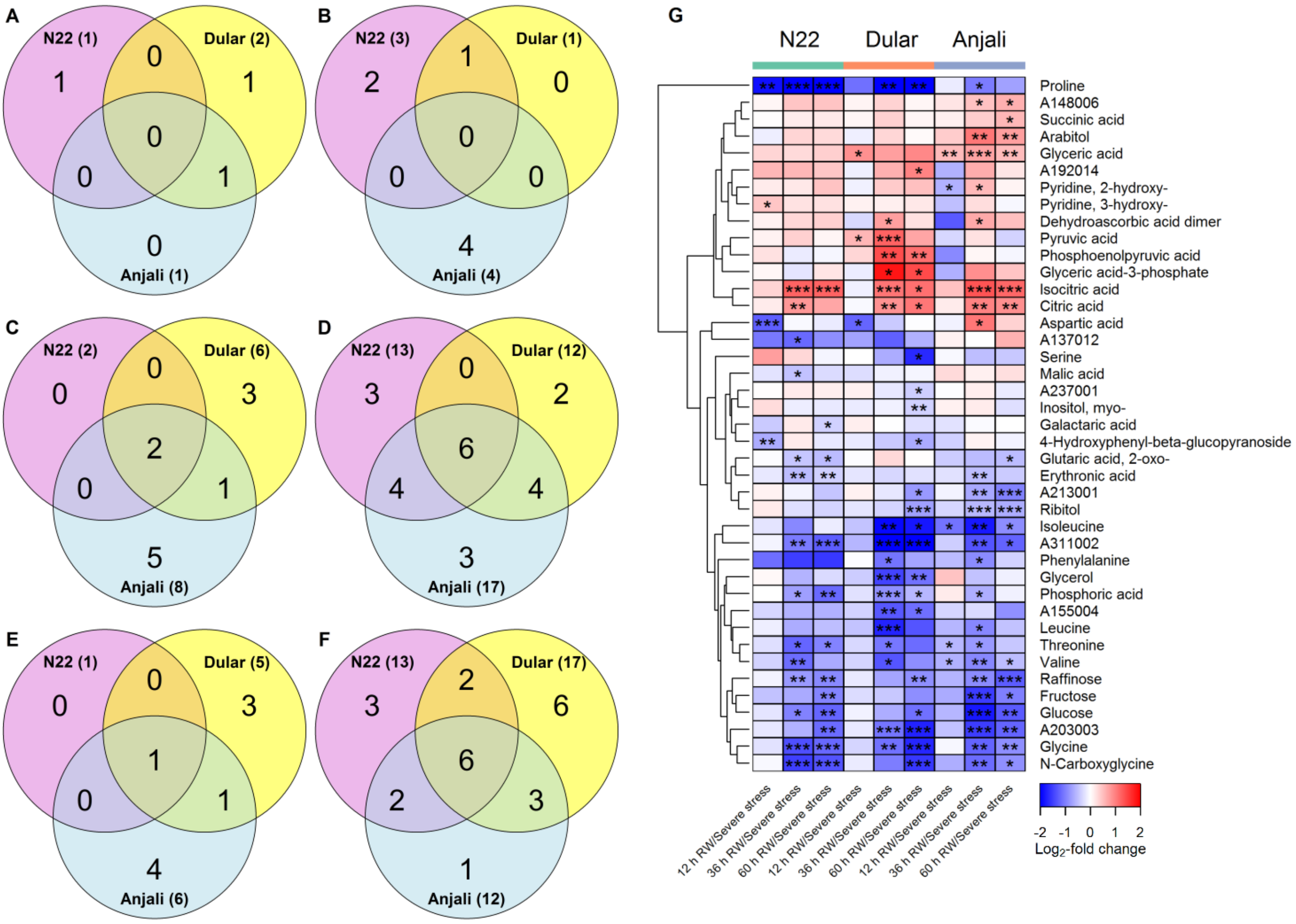

Figure 5

Click here to access/download;Figure;Figure 5_HxD_rewatering.pdf

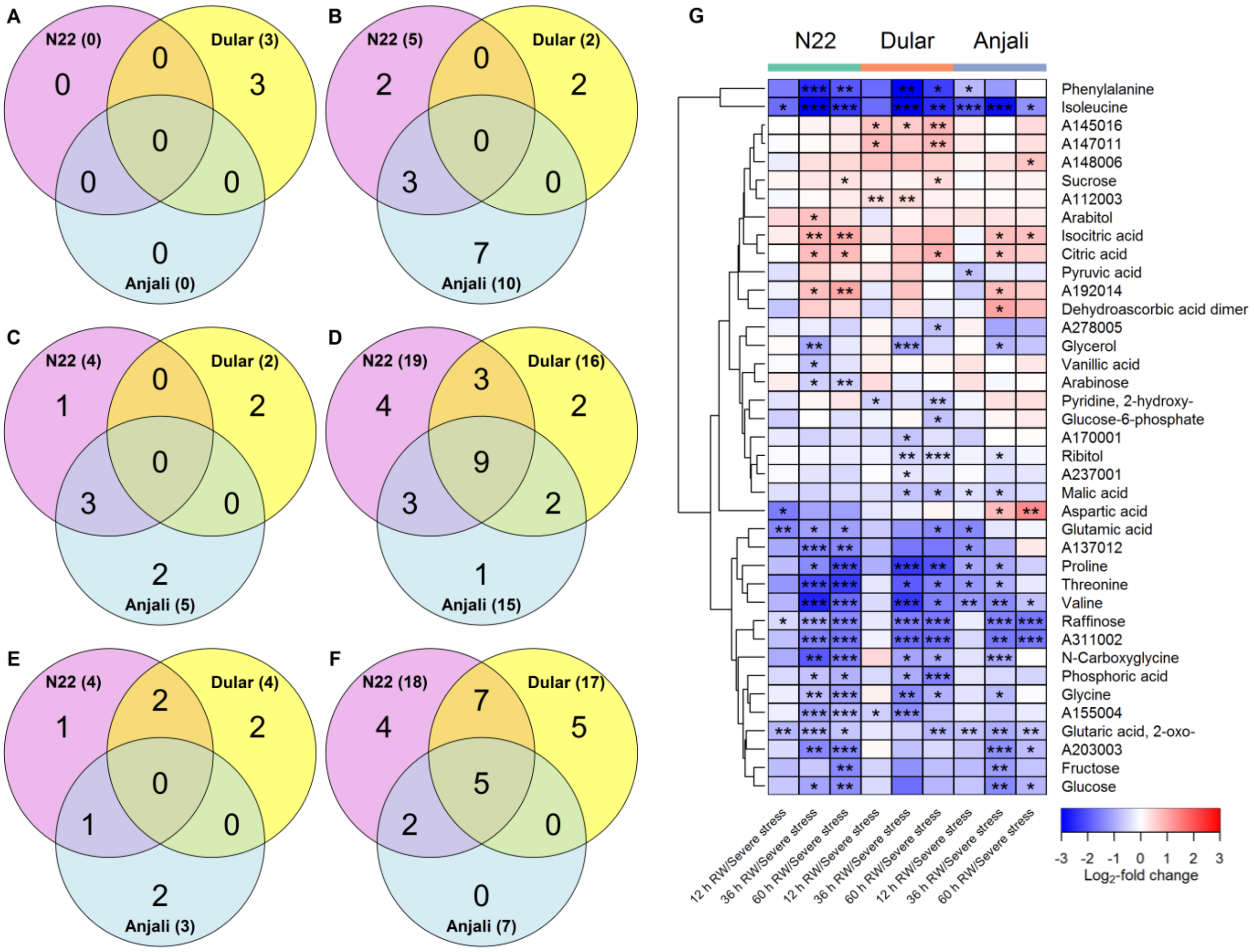

Figure 6

Click here to access/download;Figure;Figure 6_HxD_rewatering.pdf

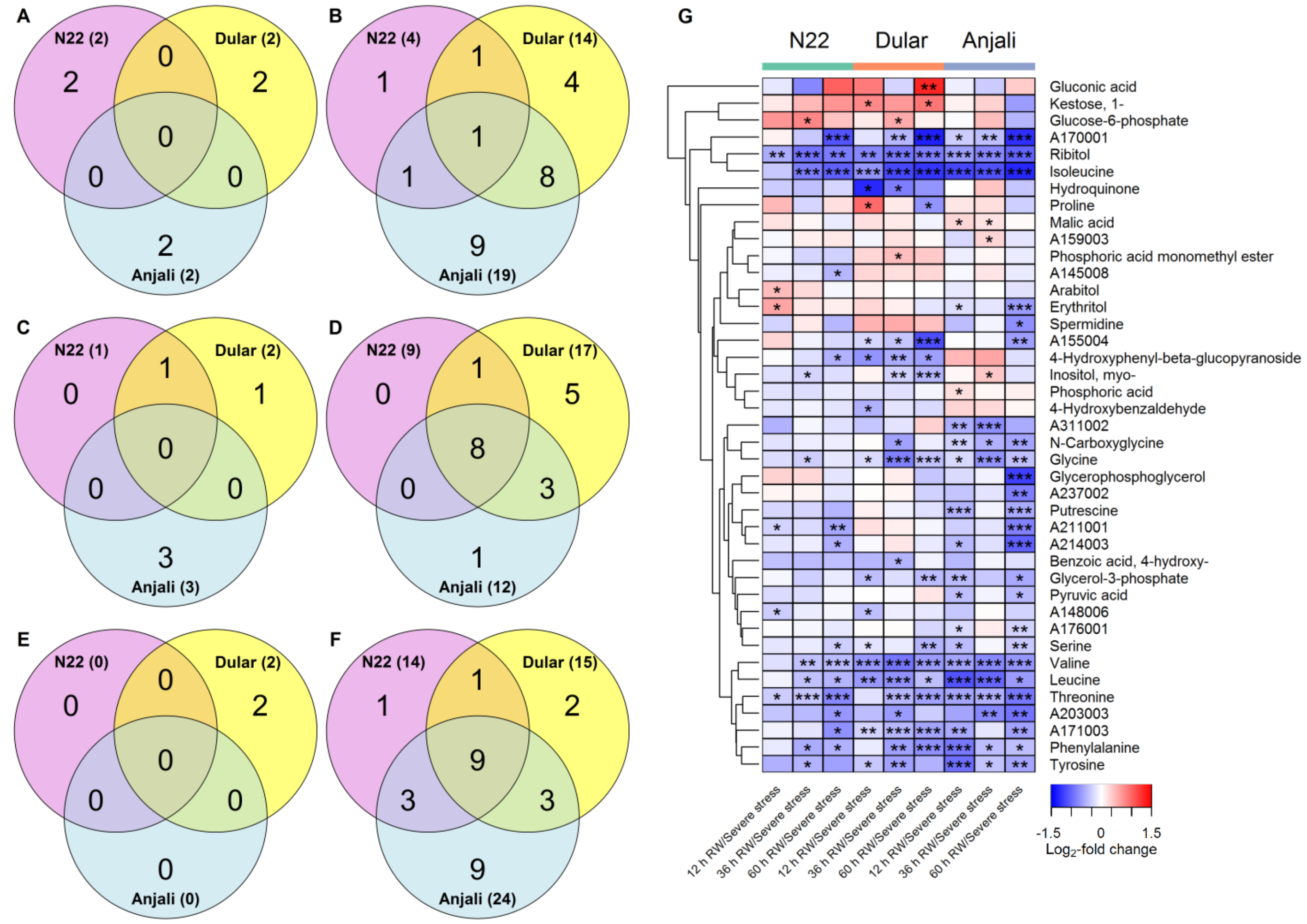

Figure 7

Click here to access/download;Figure;Figure 7_HxD_rewatering.pdf

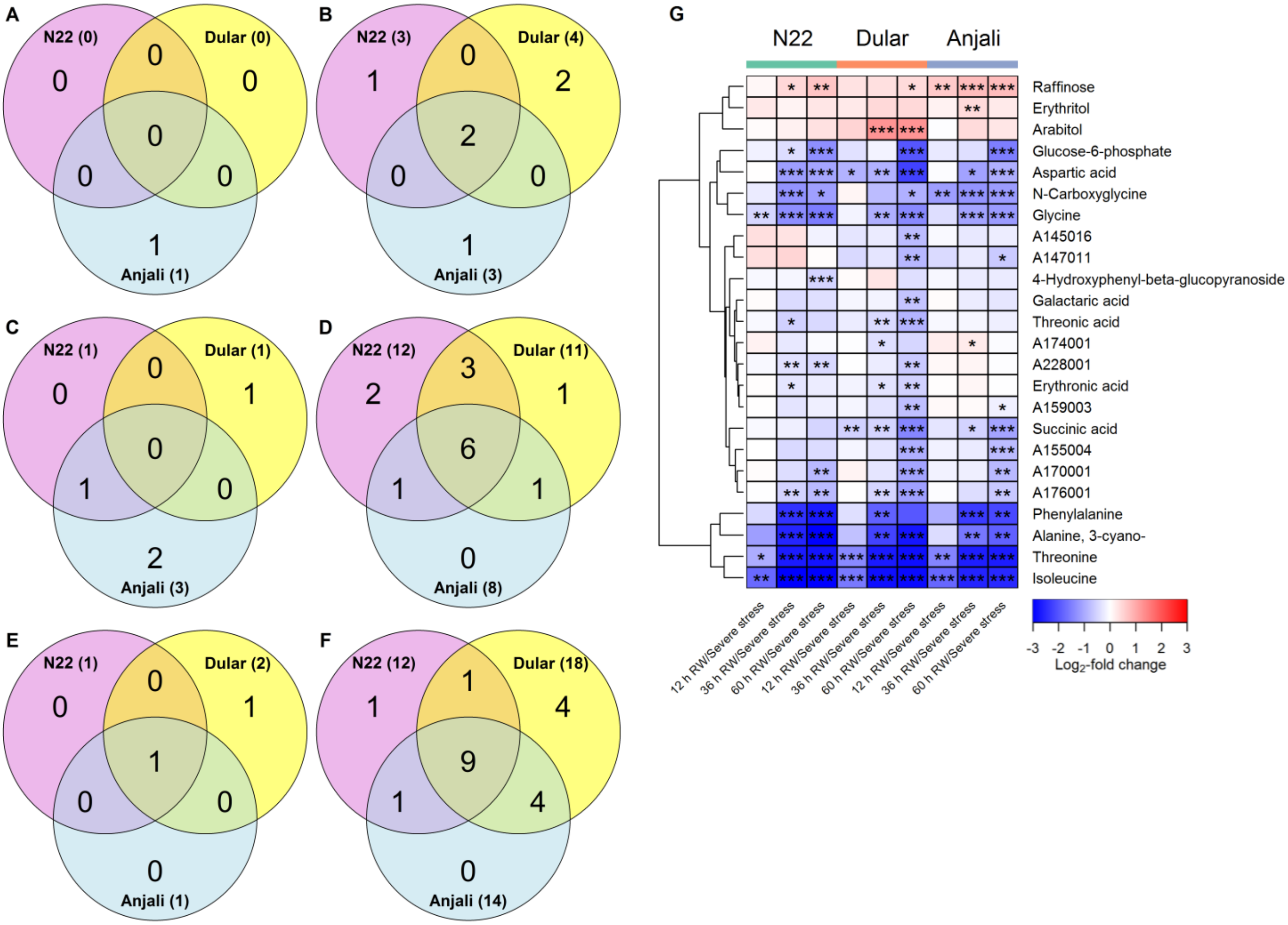

Additional file 1

Click here to access/download
Supplementary Material
Additional file 1_HxD_rewatering.pdf

Additional file 2

Click here to access/download
Supplementary Material
Additional file 2_HxD_rewatering.pdf

Additional file 3

Click here to access/download
Supplementary Material
Additional file 3_HxD_rewatering.pdf

Additional file 4

Click here to access/download
Supplementary Material
Additional file 4_HxD_rewatering.pdf

Additional file 5

Click here to access/download
Supplementary Material
Additional file 5_HxD_rewatering.pdf

Additional file 6

Click here to access/download

Supplementary Material

Additional file 6_HxD_rewatering.pdf

Additional file 7

Click here to access/download
Supplementary Material
Additional file 7_HxD_rewatering.pdf

Additional file 8

Click here to access/download
Supplementary Material
Additional file 8_HxD_rewatering.pdf
