# Supplementary figures and images for: Metabolic responses of rice source and sink organs during recovery from combined drought and heat stress in the field
Source: Gigascience. 2019 Aug 21;8(8):giz102. doi: 10.1093/gigascience/giz102 (PMC6703437; doi:10.1093/gigascience/giz102)

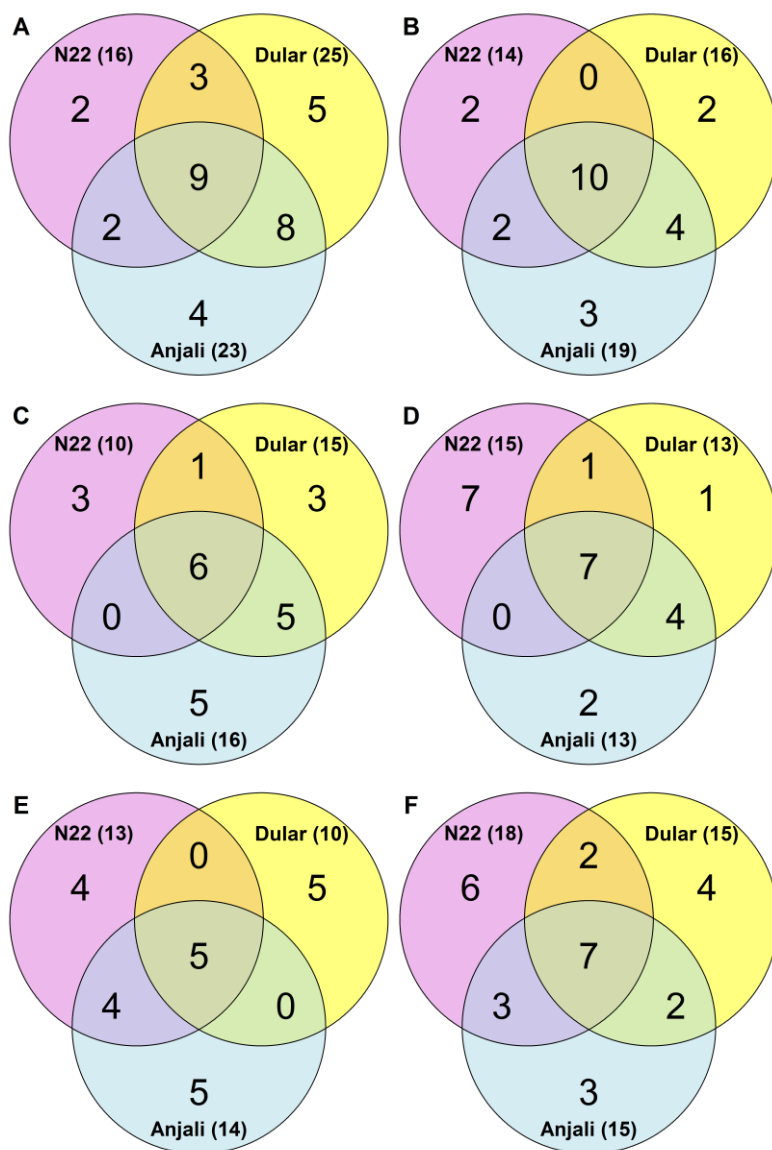

Supplement: giz102_Supplemental_Files [file giz102_supplemental_files.zip › Additional file 1_HxD_rewatering.pdf]

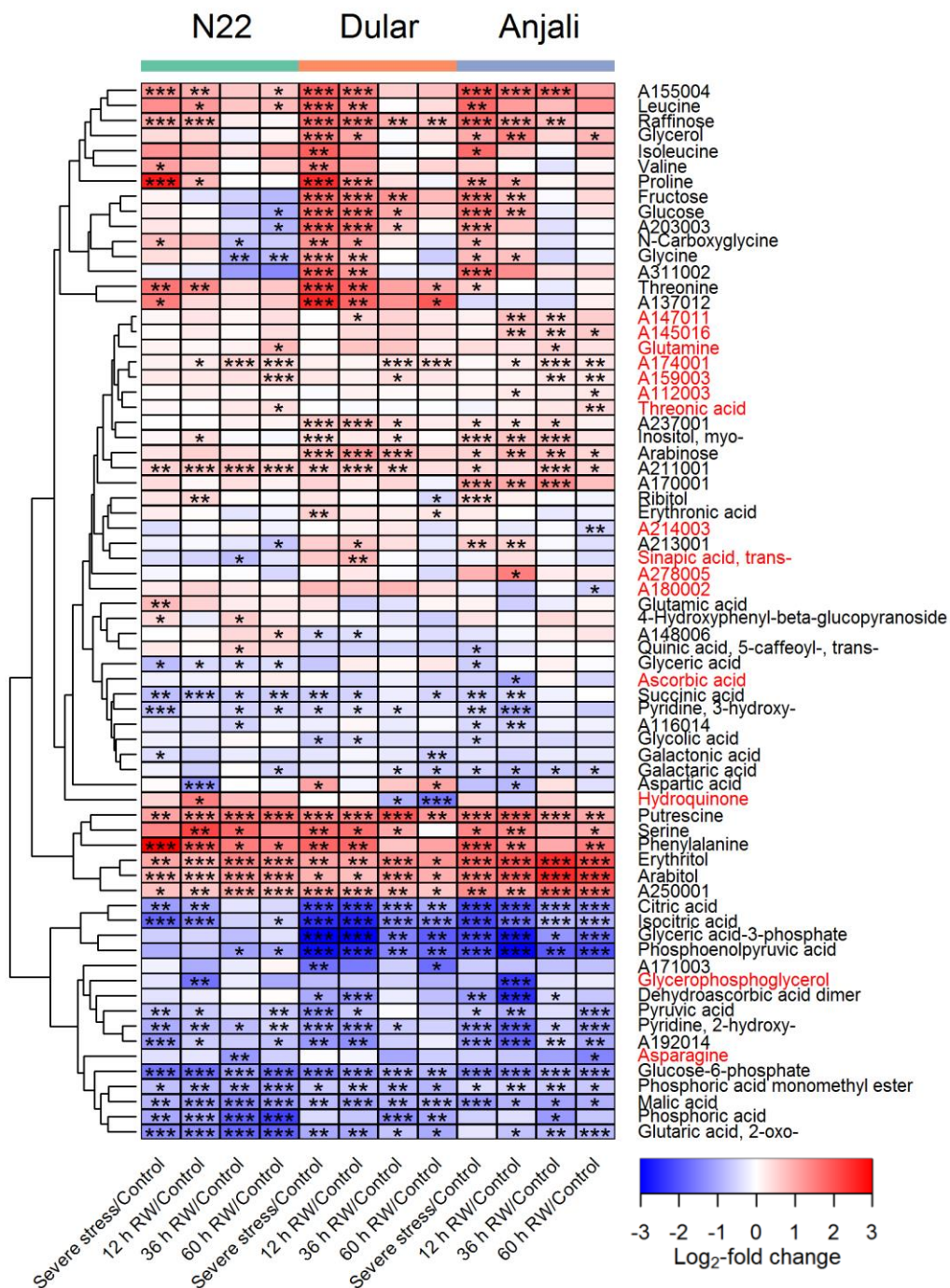

Supplement: giz102_Supplemental_Files [file giz102_supplemental_files.zip › Additional file 2_HxD_rewatering.pdf]

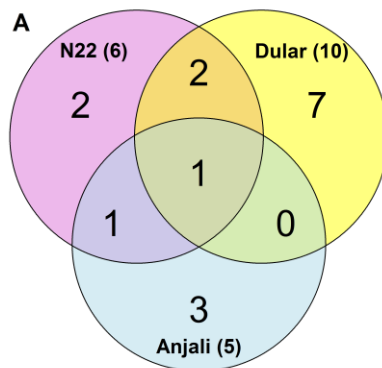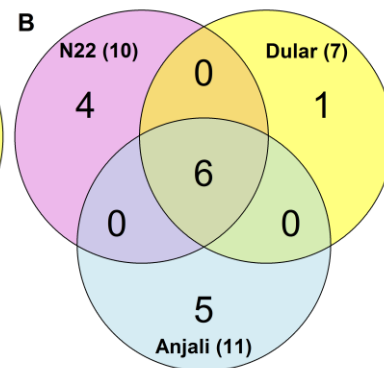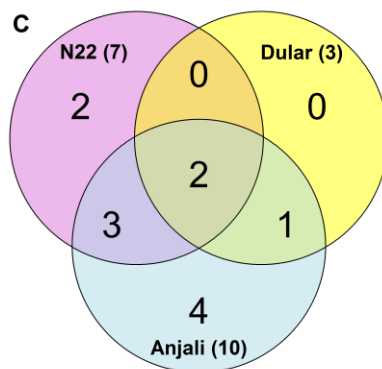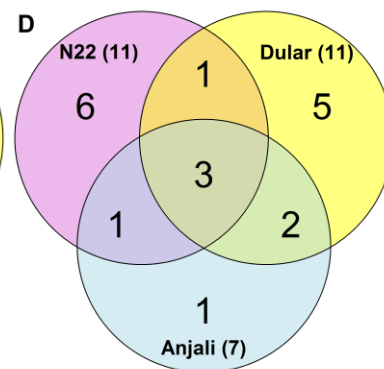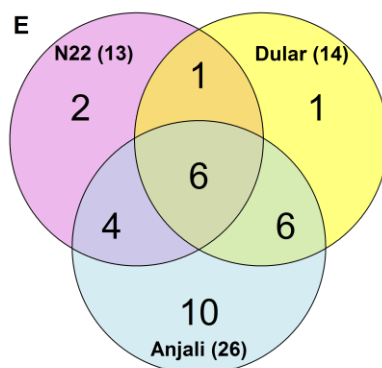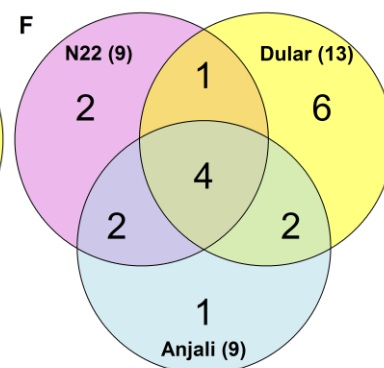

Supplement: giz102_Supplemental_Files [file giz102_supplemental_files.zip › Additional file 3_HxD_rewatering.pdf]

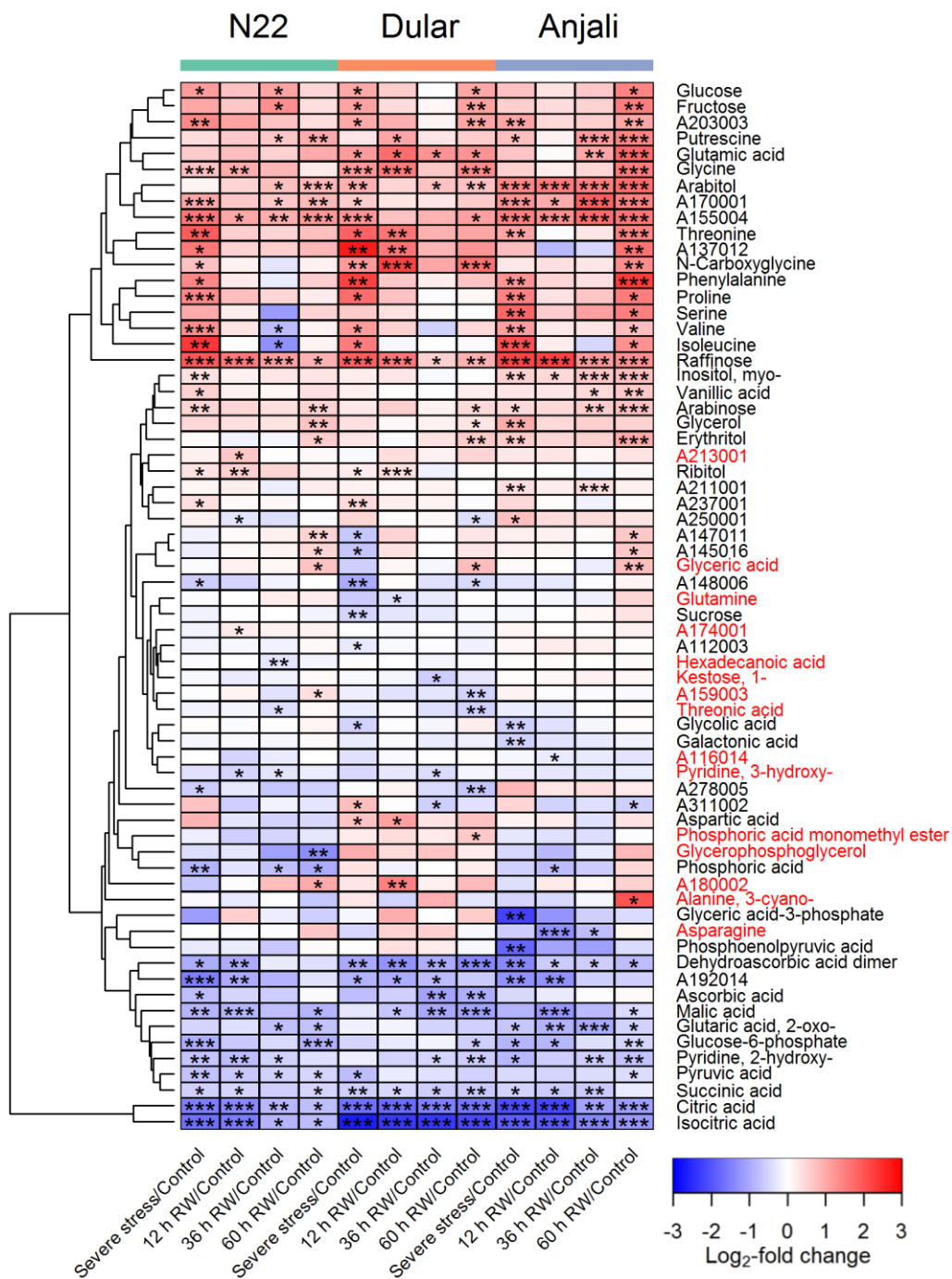

Supplement: giz102_Supplemental_Files [file giz102_supplemental_files.zip › Additional file 4_HxD_rewatering.pdf]

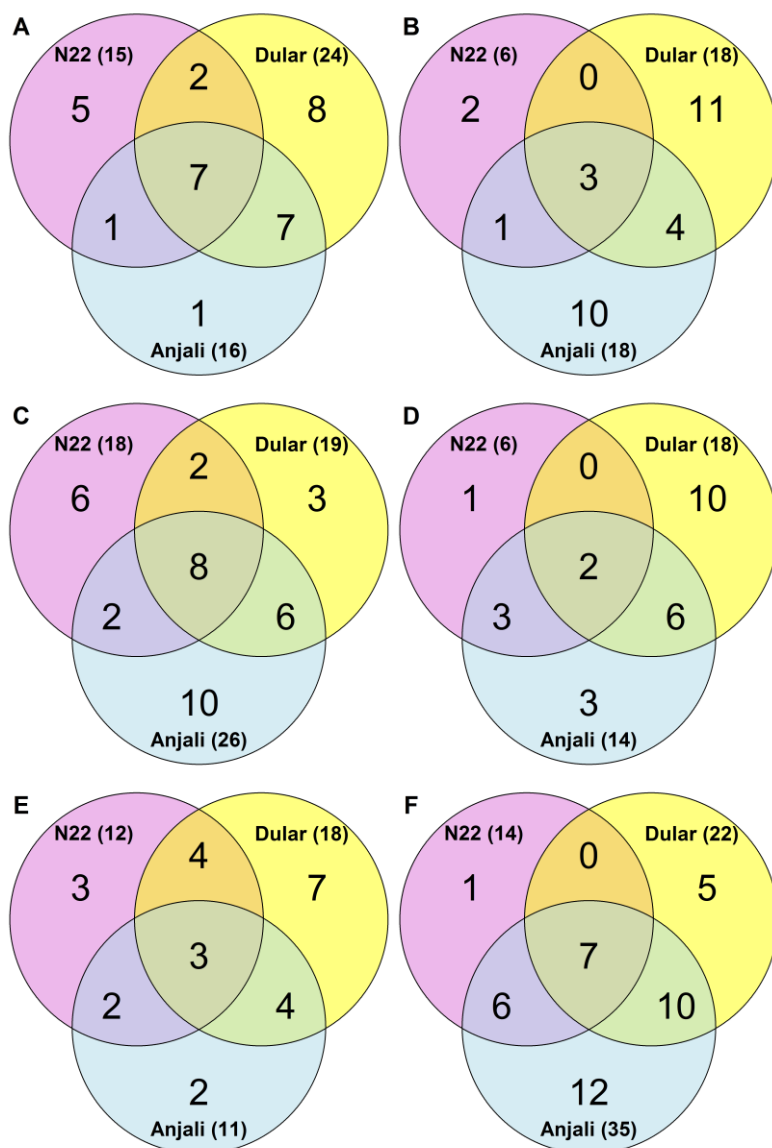

Supplement: giz102_Supplemental_Files [file giz102_supplemental_files.zip › Additional file 5_HxD_rewatering.pdf]

N22

Dular

Anjali

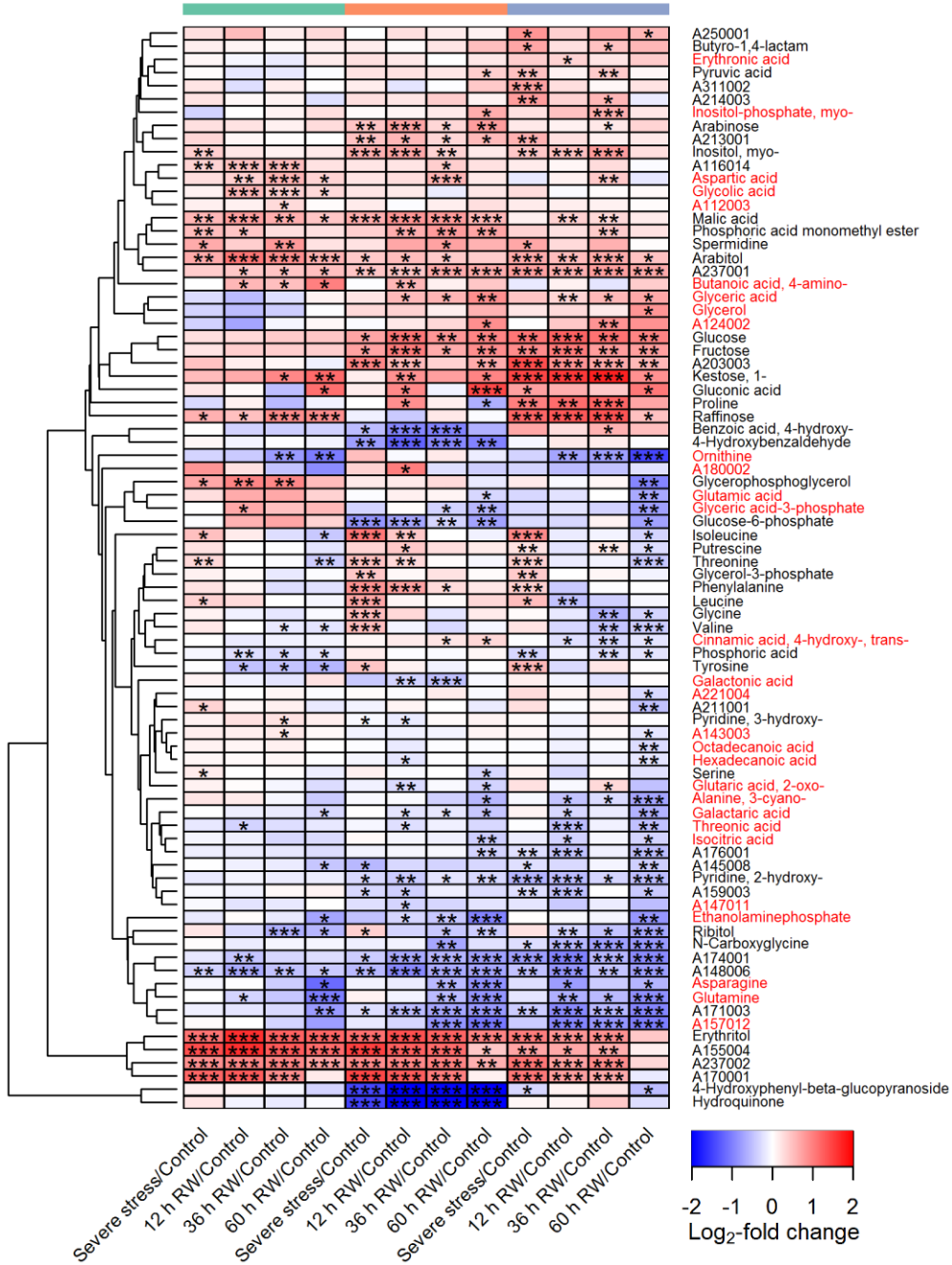

Supplement: giz102_Supplemental_Files [file giz102_supplemental_files.zip › Additional file 6_HxD_rewatering.pdf]

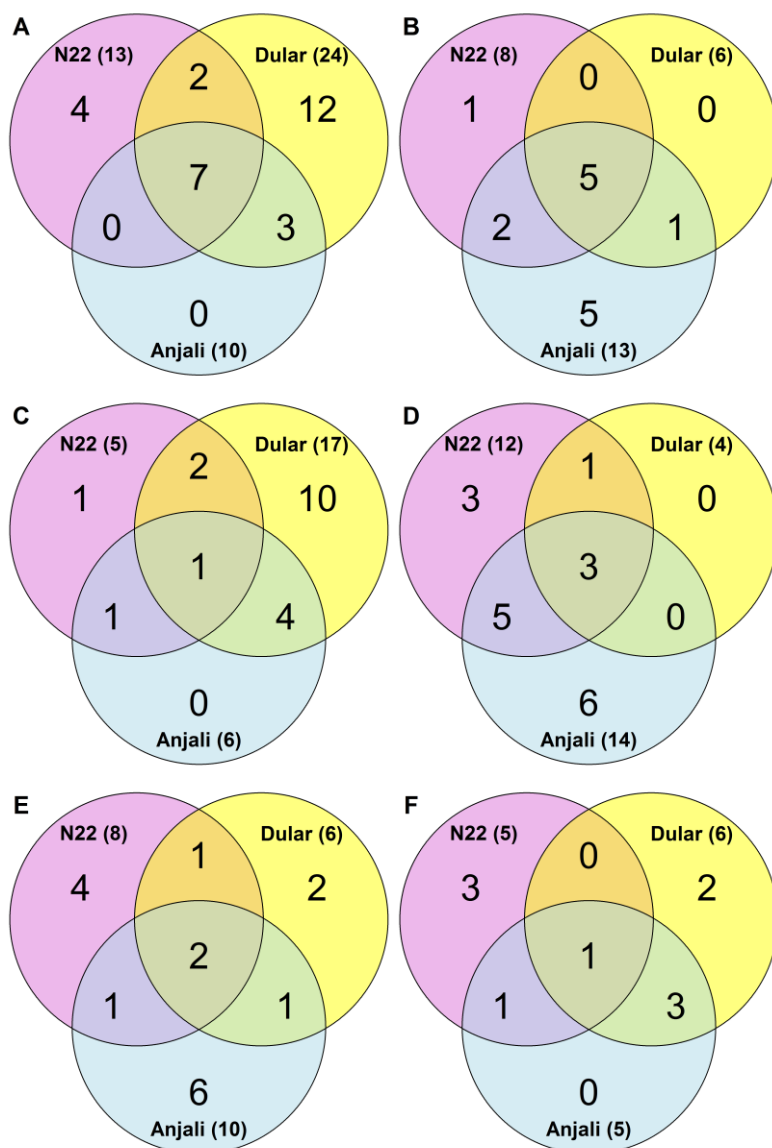

Supplement: giz102_Supplemental_Files [file giz102_supplemental_files.zip › Additional file 7_HxD_rewatering.pdf]

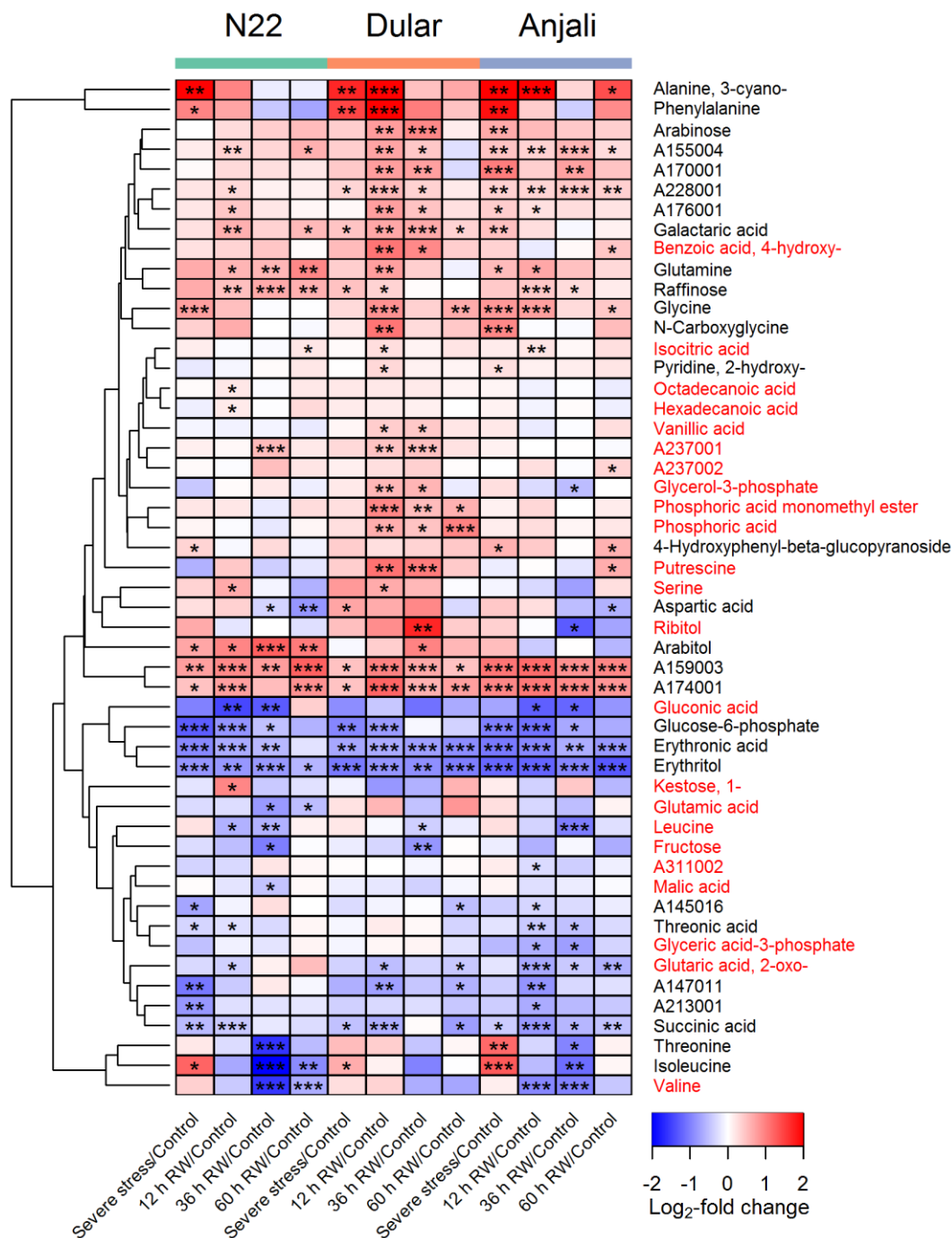

Supplement: giz102_Supplemental_Files [file giz102_supplemental_files.zip › Additional file 8_HxD_rewatering.pdf]
