# Supplementary material for: Metabolic responses of rice source and sink organs during recovery from combined drought and heat stress in the field
Source: Gigascience. 2019 Aug 21;8(8):giz102. doi: 10.1093/gigascience/giz102 (PMC6703437; doi:10.1093/gigascience/giz102)
Supplement: giz102_Response_to_Reviewer_Comments_Original_Submission [file giz102_response_to_reviewer_comments_original_submission.pdf]

Author's Response To Reviewer Comments

Close

Reviewer reports:
Reviewer #1: 1) Are the methods appropriate to the aims of the study, are they well described, and are necessary controls included?

Experimental rational, methods, scripts and datasets are well descibed and links provided for the research community. Overall, an exellent manuscript for an important research question and the authors have a novel approach. The complexity of metabalomic data (and selection of adequate controls) makes interperation of this type of data extremely difficult. However, the authors address these concerns in their discussion, tempered expectations, and have not over interperated their results.

2) Are the conclusions adequately supported by the data shown?

The conclusions the authors have drawn are supported by their data and the statistical analysis they have performed. Further work as suggested by the authors to examine the effect of these metabolites in response to combined heat and drought stress. Both heat and drought are complex abiotic stresses and I would expect metabalomic results to vary, especially when working in field across years rather than under controlled enviroments.

3) Please indicate the quality of language in the manuscript. Does it require a heavy editing for language and clarity?

The manuscript is clear and well written.
Only one mistake I could find during proof reading:
377 stress. Obviously, {T}his hypothesis needs further testing with a larger panel of genotypes.
Response: We thank the reviewer for the careful reading and for pointing this out. We have made the necessary correction.

4) Are you able to assess all statistics in the manuscript, including the appropriateness of statistical tests used?

From the description in this manuscript, previous experience with PCA and brief look at the source code provided, the analysis seems solid. More documentation in the source code would excellent for others doing similar work.

Recommendation:

This manuscript is well founded and well written. The lack of similar studies (as described by the authors) warrents the acceptance of this paper for the research community.
This manuscript is of interest to others working in metabolomics in under field conditions and would stimulate the use of this approach into field trials (If this approach can be scaled up).

Reviewer #2: The manuscript titled "Metabolic responses of rice source and sink organs during recovery from combined drought and heat stress in the field" investigated the plant recovery mechanisms after drought and heat stress. The study aim is clear and well designed. The data analysis is rigorous, and the conclusion drawn are supported from the results.
Nevertheless, I would suggest reviewing and modify some details that are given below:

Abstract
- Line 43-44 and line 56: the authors stated that the identified metabolites might be useful to ensure FOOD SECURITY under climate changing conditions. According to the FAO definition, food security encompasses different aspects related to food, such as availability, access, utilization, stability and safety. However, I think that the results of this study are useful only in ensuring food availability under

climate changing conditions, there is no improvement from the safety point of view. Therefore, I would suggest changing "food security" for "food availability".
Response: We agree with the reviewer and have changed the phrase as suggested in both places.

Data description
- Line 108-111: I would suggest moving this paragraph to the result section
Response: We would rather leave this information under Data description. We think that it is important at that point in the paper, because it provides the reader with the background necessary to understand the rational of the experiments. Also, these are not new results, we simply cite an earlier paper.

- Line 112: the authors stated that overall they collected 1241 samples, but I cannot understand how to get this number. How many samples/per years? How many samples/per time points? How many biological replicates?
Response: We have included a more detailed description of the sample and replicate numbers in Methods/Sample collection now to clarify this point.

Analysis and discussion:
- Line 134: Did the author observed any influence of the harvesting years on the PCA plot?
Response: There were differences in metabolite composition between years as conditions in the field are never the same in different years. However, we did not analyse these differences further, but rather treated the samples from all years as replicates to obtain robust metabolic responses. We have therefore not elaborated on the point of yearly variation in our paper.

- The authors focused on the primary metabolism changes. However, lipids are well known to be involved in the plant response to stresses. May the authors comment on that?
Response: We agree that lipids, along with secondary metabolites and other compound classes may also be of importance. However, since we have no data on these other compound classes it did not seem appropriate for us to speculate on that.

Methods
- Line 461-462: three to five replicates. What influence the number of collected replicates per sample? Did the authors analyse all the collected sample replicates?
Response: Yes, we analysed all replicates we collected. In most cases we obtained 5 replicates per year, giving us 15 replicates in total across the three years. In some cases it was 4 and in only a few cases it was 3. This is now stated explicitly under Methods/Sample collection to clarify this point. We do not think that the small fraction of samples with less than 14 replicates in total (only about 7%) had any influence on our analyses or the interpretation of the results.

Close
