# Supplementary material for: Metabolic responses of rice source and sink organs during recovery from combined drought and heat stress in the field
Source: Gigascience. 2019 Aug 21;8(8):giz102. doi: 10.1093/gigascience/giz102 (PMC6703437; doi:10.1093/gigascience/giz102)
Supplement: giz102_Reviewer_1_Report_Original_Submission — Gordon Wellman -- 5/27/2019 Reviewed [file giz102_reviewer_1_report_original_submission.pdf]

**Reviewer Report**

**Title: Metabolic responses of rice source and sink organs during recovery from combined drought and heat stress in the field**

**Version: Original Submission** **Date:** 5/27/2019

**Reviewer name: Gordon Wellman**

**Reviewer Comments to Author:**

1) Are the methods appropriate to the aims of the study, are they well described, and are necessary controls included?
Experimental rational, methods, scripts and datasets are well descibed and links provided for the research community. Overall, an exellent manuscript for an important research question and the authors have a novel approach. The complexity of metabalomic data (and selection of adequate controls) makes interperation of this type of data extremely difficult. However, the authors address these concerns in their discussion, tempered expectations, and have not over interperated their results.
2) Are the conclusions adequately supported by the data shown?
The conclusions the authors have drawn are supported by their data and the statistical analysis they have performed. Further work as suggested by the authors to examine the effect of these metabolites in response to combined heat and drought stress. Both heat and drought are complex abiotic stresses and I would expect metabalomic results to vary, especially when working in field across years rather than under controlled enviroments.
3) Please indicate the quality of language in the manuscript. Does it require a heavy editing for language and clarity?
The manuscript is clear and well written.
Only one mistake I could find during proof reading:
377 stress. Obviously, {T}his hypothesis needs further testing with a larger panel of genotypes.
4) Are you able to assess all statistics in the manuscript, including the appropriateness of statistical tests used?
From the description in this manuscript, previous experience with PCA and brief look at the source code provided, the analysis seems solid. More documentation in the source code would excellent for others doing similar work.
Recommendation:
This manuscript is well founded and well written. The lack of similar studies (as described by the authors) warrents the acceptance of this paper for the research community.
This manuscript is of interest to others working in metabolomics in under field conditions and would stimulate the use of this approach into field trials (If this approach can be scaled up).

**Methods**

Are the methods appropriate to the aims of the study, are they well described, and are necessary controls included? Choose an item.

**Conclusions**

Are the conclusions adequately supported by the data shown? Choose an item.

**Reporting Standards**

Does the manuscript adhere to the journal's guidelines on minimum standards of reporting? Choose an item.

Choose an item.

**Statistics**

Are you able to assess all statistics in the manuscript, including the appropriateness of statistical tests used? Choose an item.

**Quality of Written English**

Please indicate the quality of language in the manuscript: Choose an item.

**Declaration of Competing Interests**

Please complete a declaration of competing interests, considering the following questions:

- Have you in the past five years received reimbursements, fees, funding, or salary from an organisation that may in any way gain or lose financially from the publication of this manuscript, either now or in the future?
- Do you hold any stocks or shares in an organisation that may in any way gain or lose financially from the publication of this manuscript, either now or in the future?
- Do you hold or are you currently applying for any patents relating to the content of the manuscript?
- Have you received reimbursements, fees, funding, or salary from an organization that holds or has applied for patents relating to the content of the manuscript?
- Do you have any other financial competing interests?
- Do you have any non-financial competing interests in relation to this paper?

If you can answer no to all of the above, write 'I declare that I have no competing interests' below. If your reply is yes to any, please give details below.

I declare that I have no competing interests

I agree to the open peer review policy of the journal. I understand that my name will be included on my report to the authors and, if the manuscript is accepted for publication, my named report including any attachments I upload will be posted on the website along with the authors' responses. I agree for my

report to be made available under an Open Access Creative Commons CC-BY license (http://creativecommons.org/licenses/by/4.0/). I understand that any comments which I do not wish to be included in my named report can be included as confidential comments to the editors, which will not be published.

I agree to the open peer review policy of the journal

To further support our reviewers, we have joined with Publons, where you can gain additional credit to further highlight your hard work (see: https://publons.com/journal/530/gigascience). On publication of this paper, your review will be automatically added to Publons, you can then choose whether or not to claim your Publons credit. I understand this statement.

Yes Choose an item.
