# Supplementary material for: Metabolic responses of rice source and sink organs during recovery from combined drought and heat stress in the field
Source: Gigascience. 2019 Aug 21;8(8):giz102. doi: 10.1093/gigascience/giz102 (PMC6703437; doi:10.1093/gigascience/giz102)
Supplement: giz102_Reviewer_2_Report_Original_Submission — Laura Righetti -- 7/10/2019 Reviewed [file giz102_reviewer_2_report_original_submission.pdf]

Reviewer Report

Title: Metabolic responses of rice source and sink organs during recovery from combined drought and heat stress in the field

Version: Original Submission **Date:** 7/10/2019

Reviewer name: Laura Righetti

Reviewer Comments to Author:

The manuscript titled "Metabolic responses of rice source and sink organs during recovery from combined drought and heat stress in the field" investigated the plant recovery mechanisms after drought and heat stress. The study aim is clear and well designed. The data analysis is rigorous, and the conclusion drawn are supported from the results.
Nevertheless, I would suggest reviewing and modify some details that are given below:
Abstract
- Line 43-44 and line 56: the authors stated that the identified metabolites might be useful to ensure FOOD SECURITY under climate changing conditions. According to the FAO definition, food security encompasses different aspects related to food, such as availability, access, utilization, stability and safety. However, I think that the results of this study are useful only in ensuring food availability under climate changing conditions, there is no improvement from the safety point of view. Therefore, I would suggest changing "food security" for "food availability".
Data description
- Line 108-111: I would suggest moving this paragraph to the result section
- Line 112: the authors stated that overall they collected 1241 samples, but I cannot understand how to get this number. How many samples/per years? How many samples/per time points? How many biological replicates?
Analysis and discussion:
- Line 134: Did the author observed any influence of the harvesting years on the PCA plot?
- The authors focused on the primary metabolism changes. However, lipids are well known to be involved in the plant response to stresses. May the authors comment on that?
Methods
- Line 461-462: three to five replicates. What influence the number of collected replicates per sample? Did the authors analyse all the collected sample replicates?

Methods

Are the methods appropriate to the aims of the study, are they well described, and are necessary controls included? Choose an item.

Conclusions

Are the conclusions adequately supported by the data shown? Choose an item.

Reporting Standards

Does the manuscript adhere to the journal's guidelines on minimum standards of reporting? Choose an item.

Choose an item.

Statistics

Are you able to assess all statistics in the manuscript, including the appropriateness of statistical tests used? Choose an item.

Quality of Written English

Please indicate the quality of language in the manuscript: Choose an item.

Declaration of Competing Interests

Please complete a declaration of competing interests, considering the following questions:

- Have you in the past five years received reimbursements, fees, funding, or salary from an organisation that may in any way gain or lose financially from the publication of this manuscript, either now or in the future?
- Do you hold any stocks or shares in an organisation that may in any way gain or lose financially from the publication of this manuscript, either now or in the future?
- Do you hold or are you currently applying for any patents relating to the content of the manuscript?
- Have you received reimbursements, fees, funding, or salary from an organization that holds or has applied for patents relating to the content of the manuscript?
- Do you have any other financial competing interests?
- Do you have any non-financial competing interests in relation to this paper?

If you can answer no to all of the above, write 'I declare that I have no competing interests' below. If your reply is yes to any, please give details below.

I declare that I have no competing interests

I agree to the open peer review policy of the journal. I understand that my name will be included on my report to the authors and, if the manuscript is accepted for publication, my named report including any attachments I upload will be posted on the website along with the authors' responses. I agree for my report to be made available under an Open Access Creative Commons CC-BY license (http://creativecommons.org/licenses/by/4.0/). I understand that any comments which I do not wish to be included in my named report can be included as confidential comments to the editors, which will not be published.

I agree to the open peer review policy of the journal

To further support our reviewers, we have joined with Publons, where you can gain additional credit to further highlight your hard work (see: https://publons.com/journal/530/gigascience). On publication of this paper, your review will be automatically added to Publons, you can then choose whether or not to claim your Publons credit. I understand this statement.

Yes Choose an item.
